# Optimal lifestyle patterns for delaying ageing and reducing all-cause mortality: insights from the UK Biobank

**DOI:** 10.1186/s11556-024-00362-7

**Published:** 2024-10-05

**Authors:** Ce Liu, Zhaoru Yang, Li He, Ya Xiao, Hao Zhao, Ling Zhang, Tong Liu, Rentong Chen, Kai Zhang, Bin Luo

**Affiliations:** 1 Institute of Occupational Health and Environmental Health, School of Public Health, Lanzhou University, Lanzhou, People’s Republic of China; 2 Department of Environmental Health Sciences, School of Public Health, University at Albany, State University of New York, Rensselaer, New York, United States

**Keywords:** Biological aging, Lifestyle patterns, All-cause mortality, Energy-adjusted dietary inflammatory index

## Abstract

**Background:**

With the rapid aging of the global population, identifying lifestyle patterns that effectively delay aging and reduce mortality risk is of paramount importance. This study utilizes the UK Biobank to analyze the associations of the Dietary Inflammatory Index, physical activity, and sleep on biological aging and all-cause mortality.

**Methods:**

A prospective cohort study was conducted using data from over half a million UK Biobank participants. Two datasets were created by subjective and objective measurements of physical activity: the Subjective Physical Activity (SPA) and Objective Physical Activity (OPA) datasets. Lifestyle patterns, including diet habits, exercise levels, and sleep quality, were assessed within these datasets. Biological aging was quantified using validated methods, including Homeostatic Dysregulation, Klemera-Doubal Method Biological Age, Phenotypic Age, and Telomere Length. All-cause mortality data were obtained from the National Health Service. Statistical analyses included weighted linear regression and Cox proportional hazard models, adjusted for a range of covariates.

**Results:**

The findings indicate that, in most cases, maintaining an anti-inflammatory diet, engaging in at least moderate physical activity, and ensuring healthy sleep conditions are associated with delayed physiological aging (Cohen’s d ranging from 0.274 to 0.633) and significantly reduced risk of all-cause mortality (HR-SPA: 0.690, 95% CI: 0.538, 0.884; HR-OPA: 0.493, 95% CI: 0.293, 0.828). These effects are particularly pronounced in individuals under 60 years of age and in women. However, it was observed that the level of physical activity recommended by the World Health Organization (600 MET-minutes/week) does not achieve the optimal effect in delaying biological aging. The best effect in decelerating biological aging was seen in the high-level physical activity group (≥ 3000 MET-minutes/week). The study also highlights the potential of biological age acceleration and telomere length as biomarkers for predicting the risk of mortality.

**Conclusions:**

Choosing healthy lifestyle patterns, especially an anti-inflammatory diet, at least moderate physical activity, and healthy sleep patterns, is crucial for delaying aging and reducing mortality risk. These findings support the development of targeted interventions to improve public health outcomes. Future research should focus on objective assessments of lifestyle to further validate these associations.

**Supplementary Information:**

The online version contains supplementary material available at 10.1186/s11556-024-00362-7.

## Introduction

As the global population ages, the proportion of individuals aged 60 and over is projected to rise from 12% in 2015 to 22% by 2050, nearly doubling [1]. Identifying a lifestyle pattern that effectively delays ageing and reduces the risk of mortality for the majority is of paramount importance. The association between lifestyle patterns and biological ageing has been a subject of extensive research, particularly in the context of its implications for all-cause mortality. The intricate relationship between these factors is pivotal in understanding the mechanisms that underpin ageing and the potential for lifestyle modifications to mitigate its effects [2].

Biological ageing is a complex, multifactorial process influenced by an array of lifestyle choices, from dietary habits to levels of physical activity, as well as genetic and environmental factors [3, 4]. The cumulative impact of these choices can either accelerate or decelerate the ageing process at the cellular level, thereby influencing the overall health trajectory of an individual [5]. The concept of ‘biological age’, as opposed to chronological age, provides a more nuanced understanding of an individual’s health status and susceptibility to age-related diseases [6].

The UK Biobank, with its vast and diverse participant cohort, presents a unique opportunity to analyze the impact of various lifestyle patterns on biological ageing. By integrating multidimensional data, including genetics, metabolomics, and longitudinal health records, we aim to dissect the complex interplay between lifestyle factors such as diet intake, exercise, and sleep quality, and ageing. Furthermore, we seek to determine the lifestyle configurations most conducive to longevity and health.

The interplay between lifestyle factors and biological aging has garnered significant attention in recent years. Studies have demonstrated associations between individual lifestyle components—such as diet, physical activity, and sleep patterns—and various markers of biological aging and mortality risk [7–9]. Anti-inflammatory diets have been linked to reduced inflammatory markers and lower mortality risk [10], while regular physical activity has been associated with longer telomeres and slower biological aging [11, 12]. Similarly, optimal sleep duration and quality have shown correlations with better health outcomes and longevity [13, 14].

Despite these insights, significant gaps remain in our understanding of how these lifestyle factors collectively influence biological aging and mortality risk. Few studies have comprehensively examined the combined effects of diet, physical activity, and sleep on multiple markers of biological age. Moreover, the optimal levels of these lifestyle factors for delaying aging and reducing mortality risk are yet to be fully elucidated.

This study aims to address these knowledge gaps by leveraging data from the UK Biobank. We seek to investigate the associations of combined lifestyle patterns with multiple markers of biological age, determine optimal levels of these factors for delaying aging and reducing mortality risk, and explore potential variations in these associations across different demographic and health subgroups. We hypothesize that anti-inflammatory diets, higher physical activity levels, and healthier sleep patterns will be associated with lower biological age and reduced mortality risk, with their combined effect exceeding individual contributions.

## Methods

### Study design and participants

This investigation utilized the UK Biobank resource, under application number 116,198. The UK Biobank, a large-scale prospective cohort study, encompasses over half a million individuals aged 40–69, residing within proximity to 22 assessment centres across the UK. Recruitment was based on the National Health Service General Practitioner registry, with baseline assessments occurring from 2006 to 2010 [15]. A subset of 103,659 participants also engaged in an accelerometer-based physical activity study between 2013 and 2015 [16]. The main selection criteria for this study included: (1) UK Biobank participants aged 40–69 years at baseline. (2) Availability of dietary data from at least one 24-hour recall assessment. (3) Complete data on physical activity (subjective or objective) and sleep patterns. (4) Availability of biomarkers necessary for calculating biological age measures. (5) No missing data on key covariates. (6) Participants with extreme energy intake values or less than three days of accelerometer wear time were excluded. Detailed exclusion criteria are presented in Supplementary Figure S1.1. The study received ethical clearance from the North West Multi-centre Research Ethics Committee, with informed consent obtained from all subjects [17].

### Life patterns

In our study, we delineated 18 distinct lifestyle patterns by evaluating participants’ dietary habits, physical activity levels, and sleep quality (Table 1).

**Table 1:** Life pattern combinations, by combining the three (or two) classifications of diet, physical activity and sleep with each other to form 18 life patterns

Indicator	Least healthy	→	Healthiest
Energy-adjusted dietary inflammation index	Very/moderately anti-inflammatory ( < − 1)	Neutral (from − 1 to 1)	Very/moderately pro-inflammatory (> 1)
Subjective physical activity measurement ^a^	Low (< 600 MET-min/week)	Moderate (600 − 3000 MET-min/week)	High (> 3000 MET-min/week)
Objective physical activity measurement ^b^	Low (< 24.0 milli-gravitational (mg) units)	Moderate (24.0-30.4 mg)	High (> 30.4 mg)
Sleep condition	Unhealthy (sleep assessment score < 4)		Healthy (sleep assessment score ≥ 4)

^a^ Subjective physical activity measures are only available for the subjective physical activity dataset

^b^ Objective physical activity measures are only applicable to the Objective Physical Activity dataset

#### Assessment of dietary conditions

To assess dietary conditions, we calculated the energy-adjusted Dietary Inflammatory Index (E-DII) using 24-hour dietary recall data obtained through online follow-up [18]. The UK Biobank employed the Oxford WebQ, a web-based tool, to collect information on the consumption of 206 food items and 32 beverages within the past 24 h [19]. Energy and nutrient intake were computed using the McCance and Widdowson’s Composition of Foods, 5th Edition [20]. To ensure data representativeness and stability, we averaged five 24-hour recalls collected between April 2009 (initial assessment) and June 2012 (final assessment). Participants with abnormal energy intake (*n* = 2,124) were excluded based on predefined thresholds for males (< 800 kcal/day or > 4200 kcal/day) and females (< 500 kcal/day or > 3500 kcal/day) [21]. See Supplementary Material: Supplementary method 1 for specific E-DII calculations.

The theoretical range of individual E-DII scores spans from − 3.95 (highly anti-inflammatory diet) to + 7.74 (highly pro-inflammatory diet). The E-DII score was treated as a continuous variable and categorized into three groups: (i) Very/Moderately Anti-inflammatory (< -1), (ii) Neutral (≥ -1 to ≤ 1), and (iii) Very/Moderately Pro-inflammatory (> 1) [22].

#### Assessment of physical activity

The evaluation of physical activity in our study is bifurcated into two distinct segments: (1) Subjective Physical Activity Assessment; (2) Objective Physical Activity Assessment. Two distinct datasets were generated from this: the subjective physical activity assessment dataset (SPA) and the objective physical activity assessment dataset (OPA).

Subjective Physical Activity Assessment: Utilizing the UK Biobank’s adapted International Physical Activity Questionnaire, we quantified total physical activity (TPA) which includes walking, moderate, and vigorous activities over the past week [23]. Participants were categorized into three activity levels: low (< 600 MET-min/week), moderate (600–3000 MET-min/week), and high (≥ 3000 MET-min/week), aligning with the 150-minute weekly moderate-intensity PA guideline [23]. The threshold of 600 MET-minutes/week corresponds to the recommended guidelines for moderate-intensity PA of 150 min per week.

Objective Physical Activity Assessment: The UK Biobank measures participants’ physical activity using the Axivity AX3 wrist-worn triaxial accelerometer (Axivity Ltd., Newcastle, UK), which participants are instructed to wear continuously for seven days [24]. Data from participants with less than three days of wear time, missing data for any one-hour period within a 24-hour cycle, or accelerometer calibration failure were excluded. We employed the mean overall acceleration, as it quantifies the total time expended across all PA intensity levels. The mean overall acceleration was categorized into tertiles within the overall distribution and estimated based on the median. For ease of interpretation, accelerometer data for each tertile were described as corresponding levels of physical activity, informed by laboratory calibration studies [25], namely low (4 min/day), moderate (10 min/day), and high (22 min/day) of brisk walking (Supplementary material: Supplementary method 2).

#### Assessment of Sleep Behavior

Sleep behavior was collected via touchscreen questionnaire by the UK Biobank. A healthy sleep score was created by amalgamating chronotype, sleep duration, insomnia symptoms, snoring, and excessive daytime sleepiness [26]. Sleep duration was categorized into three groups: short sleep (< 7 h/day), recommended sleep duration (7–8 h/day), and long sleep duration (≥ 9 h/day). Healthy sleep factors were defined as: an early chronotype (“morning” or “more morning than evening”); sleeping 7–8 h/day; reporting never/rarely or sometimes experiencing insomnia symptoms; absence of snoring; and no excessive daytime sleepiness (“never/rarely” or “sometimes”). Each sleep factor was coded as 1 if it met the healthy standard and 0 otherwise. The total healthy sleep score was derived by summing the five individual sleep factors. A higher score indicates a healthier sleep pattern. We further categorized sleep conditions into two levels: unhealthy (< 4 points), and healthy (≥ 4 points) [27].

### Measurement of biological aging

To quantify biological aging, we employed four rigorously validated methodologies: Homeostatic Dysregulation (HD), Klemera-Doubal Method (KDM) Biological Age, Phenotypic Age [28], and Telomere Length [29].

HD is computed without incorporating chronological age, based on the Mahalanobis distance of a set of biomarkers relative to a reference sample. This metric can be interpreted as the deviation of an individual’s physiological function from the reference cohort, providing a measure of systemic balance. KDM Biological Age is derived from a regression of biomarkers on actual age, offering an estimate of the KDM Biological Age that corresponds to the approximate chronological age at which an individual’s physiological functions would be considered normal within the National Health and Nutrition Examination Survey (NHANES) III cohort, which serves as the training sample. Phenotypic Age is calculated as a linear combination of chronological age and biomarker levels, weighted by coefficients derived from a Cox proportional hazards mortality model in a reference population. This age can be interpreted as the age at which the predicted risk of death corresponds to the average risk of death in NHANES III. Telomere Length was measured using quantitative PCR to determine the relative length of leukocyte telomeres in the UK Biobank. This index represents the ratio of telomere repeat copy number to single-copy gene copy number, adjusted for the influence of technical parameters. Subsequently, it underwent logarithmic transformation to achieve normal distribution, followed by Z-standardization across the distribution of all individuals, thus measuring telomere length [30].

The computation of biological age was executed using the “BioAge” package, with detailed methodologies delineated in Supplementary Material: Supplementary method 3.

### All-cause mortality data

The all-cause mortality data were sourced from the National Health Service (NHS) Information Centre for England and Wales, and the NHS Central Register for Scotland. This work utilized patient-provided data, collected by the NHS as part of their care and support services. The survivor data for England and Wales were reviewed on November 30, 2023, and the data for Scotland were reviewed on December 31, 2023 [31].

### Covariate

Covariates were selected based on literature [32, 33]. Socioeconomic status was inferred from the Townsend deprivation index. Baseline age and gender were ascertained at initial assessment. Employment status was classified as employed, retired, or other. Education was categorized as high (degree level), intermediate (A/AS level or equivalent), or low (below A/AS level). Ethnicity encompassed Black, Chinese, Mixed, South Asian, White, and Other, as self-reported. Smoking status included three categories: current smokers, former smokers, and never smokers. Body Mass Index (BMI) was constructed based on height and weight measured during the initial assessment center visit. Alcohol intake frequency was categorized from high to low as: daily or almost daily, three to four times per week, once or twice per week, one to three times per month, only on special occasions, never. Based on previous studies on multimorbidity among UK Biobank participants [33, 34], we considered 36 chronic conditions and categorized participants into three groups (Supplementary Material: Table S1.4): no disease (none of the 36 chronic conditions), single disease (one of the 36 chronic conditions), and multimorbidity (two or more of the 36 chronic conditions). Consideration was also given to the assessment centre at the time of joining the UK Biobank. Further details can be found in Supplementary Material: Table S1.2.

### Statistical analysis

Baseline characteristics of participants were described as means (SD) for continuous variables and counts (percentage) for categorical variables. Z-standardization was applied to HD, KDM Biological Age acceleration, phenotypic age acceleration, and telomere length. Lifestyle patterns’ reference group included pro-inflammatory diets, low physical activity, and poor sleep conditions.

(i) Due to the imbalance in the distribution among different lifestyle pattern groups (Supplementary Material: Figure S3.1 and Figure S3.2), we employed weighted linear regression with the inverse of group frequencies as weights to study the impact of lifestyle patterns on biological age acceleration. Cohen’s d was used to assess the effect size of each lifestyle pattern group on the biological age acceleration [35]. Subsequently, stratified analyses were conducted across different genders, ages, and chronic disease statuses. To account for the non-linear effects of age on aging speed, non-linear terms of age were included in all models to capture these effects.
(ii) We utilized Cox proportional hazards models to estimate the hazard ratios (HRs) and their 95% confidence intervals (CI) for biological age acceleration in relation to all-cause mortality. Two types of data were considered: continuous biological age residuals and categorical biological age residuals (|Biological Age Acceleration| ≤ 1, Biological Age Acceleration < − 1, Biological Age Acceleration > 1). Two models were constructed to test the association between lifestyle patterns and all-cause mortality risk: Model 1 adjusted for age and gender; Model 2 further adjusted for all previously mentioned covariates.
(iii) Cox proportional hazards models were also used to estimate the HRs and their 95% CI for each lifestyle pattern group in relation to all-cause mortality. Stratified analyses were again performed for different genders, ages, and chronic disease statuses. We further assessed potential non-linear relationships between E-DII scores, physical activity, sleep condition scores, and all-cause mortality risk using restricted cubic splines, with the smallest Akaike Information Criterion suggesting the number and placement of knots.

In all Cox proportional hazards models, no violations of the proportional hazards assumption were observed when evaluated using Schoenfeld residuals (*P* > 0.05). All analyses were conducted in R version 4.4.3. To account for multiple testing (18 lifestyle pattern groups), Benjamini-Hochberg correction was applied, and a corrected two-sided *P* < 0.05 was considered statistically significant.

## Results

Between December 19, 2006, and December 17, 2022, the subjective physical activity measurement dataset recorded 5562 deaths among 105,705 individuals during a median follow-up of 13.47 years (IQR 12.89–14.20; 1417869 person-years), which were included in the lifestyle-associated analysis. The objective physical activity measurement dataset comprised 42,006 participants, with 1580 deaths recorded over a median follow-up period of 13.6 years (IQR 12.98–14.29; 571591.9 person-years). Baseline characteristics of the study population are displayed in Table 2.

**Table 2:** Characteristics of the UK Biobanking population for the subjective physical activity measurement dataset (*n* = 105705) and the Objective Physical Activity Measurement dataset (*n* = 42006)

	Subjective physical activity dataset (*N* = 105705)	Objective physical activity dataset (*N* = 42006)
Age (mean (SD))	55.69 (7.95)	55.95 (7.79)
Sex = Male (%)	49,312 (46.7)	18,291 (43.5)
Townsend deprivation index (mean (SD))	-1.69 (2.80)	-1.80 (2.75)
E-DII group ^a^ (%)		
Pro-inflammatory	9475 (9.0)	3392 (8.1)
Neutral	79,580 (75.3)	32,579 (77.6)
Anti-inflammatory	16,650 (15.8)	6035 (14.4)
Physical activity group ^b^ (%)		
Low	16,238 (15.4)	13,592 (32.4)
Medium	57,939 (54.8)	14,207 (33.8)
High	31,528 (29.8)	14,207 (33.8)
Sleep group ^c^ (%)		
Unhealthy	37,717 (35.7)	14,333 (34.1)
Healthy	67,988 (64.3)	27,673 (65.9)
Race (%)		
Asian or Asian British	1379 (1.3)	352 (0.8)
Black or Black British	940 (0.9)	280 (0.7)
Chinese	296 (0.3)	84 (0.2)
White	101,534 (96.1)	40,736 (97.0)
Mixed	588 (0.6)	224 (0.5)
Other ethnic	968 (0.9)	330 (0.8)
Employment status (%)		
Work	61,027 (57.7)	23,819 (56.7)
Retired	28,816 (27.3)	11,681 (27.8)
Other	15,862 (15.0)	6506 (15.5)
Qualifications (%)		
Low	75,351 (71.3)	30,045 (71.5)
Middle	14,141 (13.4)	5538 (13.2)
High	16,213 (15.3)	6423 (15.3)
Frequency of alcohol consumption (%)		
Never	5898 (5.6)	2182 (5.2)
Special occasions only	9435 (8.9)	3752 (8.9)
One to three times a month	11,238 (10.6)	4436 (10.6)
Once or twice a week	26,287 (24.9)	10,461 (24.9)
Three or four times a week	27,651 (26.2)	11,204 (26.7)
Daily or almost daily	25,196 (23.8)	9971 (23.7)
Smoking state (%)		
Current	7871 (7.4)	2673 (6.4)
Previous	37,612 (35.6)	15,058 (35.8)
Never	60,222 (57.0)	24,275 (57.8)
BMI group (%)		
Underweight	571 (0.5)	261 (0.6)
Normal	39,854 (37.7)	16,959 (40.4)
Overweight	44,450 (42.1)	17,087 (40.7)
Obese	20,830 (19.7)	7699 (18.3)
Chronic disease status ^d^ (%)		
Disease-free	47,300 (44.7)	19,211 (45.7)
Single Disease	23,314 (22.1)	9256 (22.0)
Multi-disease	35,091 (33.2)	13,539 (32.2)
Homeostatic disorder (mean (SD))	2.14 (0.92)	2.14 (0.89)
KDM age acceleration (mean (SD))	-0.91 (5.73)	-1.18 (5.66)
PhenoAge acceleration (mean (SD))	-0.50 (5.04)	-0.73 (4.93)
Telomere length ^e^ (mean (SD))	0.04 (0.99)	0.06 (0.99)

^a^ E-DII: Energy-adjusted dietary inflammation index

^b^ Physical activity group: use the subjective physical activity measurement subgroup in the subjective physical activity dataset and the objective physical activity measurement subgroup in the objective physical activity dataset

^c^ Sleep groups were grouped by sleep assessment scores

^d^ We analysed 36 common chronic conditions in the UK population. Individuals without any of these conditions were categorised as the ‘disease-free’ group, those with one condition were categorised as the ‘single disease’ group, and those with two or more conditions were categorised as the ‘multi-disease’ group

^e^ Technically adjusted leukocyte telomere lengths were both log-transformed to obtain a normal distribution and Z-standardized according to the distribution of all individuals in the UK Biobank for whom telomere length measurements were performed

**Table 3:** Relationships between KDM biological age acceleration, phenotypic age acceleration, and telomere length with all-cause mortality in the UK Biobank subjective (*n* = 105705) and objective (*n* = 42006) physical activity measurement datasets

Acceleration of biological age	HR (95% CI)	
	Subjective physical activity dataset	Objective physical activity dataset
KDM-age acceleration ^a^		
Model 1		
KDM-age acceleration, continuous (per standard deviation)	1.384^***^ (1.349, 1.419)	1.402^***^ (1.338, 1.369)
KDM-age acceleration, categorical		
|KDM-age acceleration| ≤ 1	1.000	1.000
KDM-age acceleration < − 1	0.819^***^ (0.755, 0.889)	0.724^***^ (0.626, 0.838)
KDM-age acceleration > 1	1.431^***^ (1.319, 1.551)	1.257^***^ (1.086, 1.454)
Model 2		
KDM-age acceleration, continuous (per standard deviation)	1.308^***^ (1.273, 1.344)	1.296^***^ (1.232, 1.364)
KDM-age acceleration, categorical		
|KDM-age acceleration| ≤ 1	1.000	1.000
KDM-age acceleration < − 1	0.871^**^ (0.802, 0.946)	0.748^***^ (0.646, 0.866)
KDM-age acceleration > 1	1.334^***^ (1.229, 1.447)	1.213^**^ (1.048, 1.404)
Phenotypic age acceleration ^b^		
Model 1		
Phenoage acceleration, continuous (per standard deviation)	1.405^***^ (1.373, 1.438)	1.383^***^ (1.325, 1.444)
Phenoage acceleration, categorical		
|Phenoage acceleration| ≤ 1	1.000	1.000
Phenoage acceleration < − 1	0.829^***^ (0.765, 0.899)	0.761^***^ (0.656, 0.882)
Phenoage acceleration > 1	1.477^***^ (1.367, 1.597)	1.382^***^ (1.198, 1.595)
Model 2		
Phenoage acceleration, continuous (per standard deviation)	1.331^***^ (1.299, 1.364)	1.283^***^ (1.225, 1.344)
Phenoage acceleration, categorical		
|Phenoage acceleration| ≤ 1	1.000	1.000
Phenoage acceleration < − 1	0.867^***^ (0.799, 0.940)	0.807^**^ (0.696, 0.937)
Phenoage acceleration > 1	1.359^***^ (1.256, 1.469)	1.238^**^ (1.071, 1.430)
Telomere length ^c^		
Model 1 ^d^		
Telomere length, continuous (per standard deviation)	0.934^***^ (0.909, 0.961)	0.837^***^ (0.796, 0.881)
Telomere length, categorical		
|Telomere length| ≤ 1	1.000	1.000
Telomere length < − 1	1.419^***^ (1.325, 1.520)	1.289^***^ (1.127, 1.474)
Telomere length > 1	0.769^***^ (0.706, 0.839)	0.797^**^ (0.681, 0.933)
Model 2 ^e^		
Telomere length, continuous (per standard deviation)	0.946^***^ (0.919, 0.972)	0.905^***^ (0.859, 0.953)
Telomere length, categorical		
|Telomere length| ≤ 1	1.000	1.000
Telomere length < − 1	1.251^***^ (1.168, 1.340)	1.226^***^ (1.072, 1.402)
Telomere length > 1	0.861^***^ (0.790, 0.939)	0.837^*^ (0.716, 0.980)

^*^P < 0.05, ^**^P < 0.01, ^***^P < 0.001

^a^ Refers to the difference between KDM age and actual age

^b^ Refers to the difference between Phenotypic age and actual age

^c^ Technically adjusted leukocyte telomere lengths were both log-transformed to obtain a normal distribution and Z-standardized according to the distribution of all individuals in the UK Biobank for whom telomere length measurements were performed

^d^ Model 1 adjusts for age and gender. The non-linear term for age was adjusted for models where there was a non-linear effect of age

^e^ Model 2 adjusted for age, sex, dietary inflammatory index, physical activity, sleep, Townsend deprivation index, employment status, educational qualification, ethnicity, smoking status, frequency of alcohol consumption, body mass index, UK Biobank Reception Centre, and disease status. The non-linear term for age was adjusted for models where there was a non-linear effect of age

### Optimal life patterns for delaying biological ageing

**Fig. 1:**
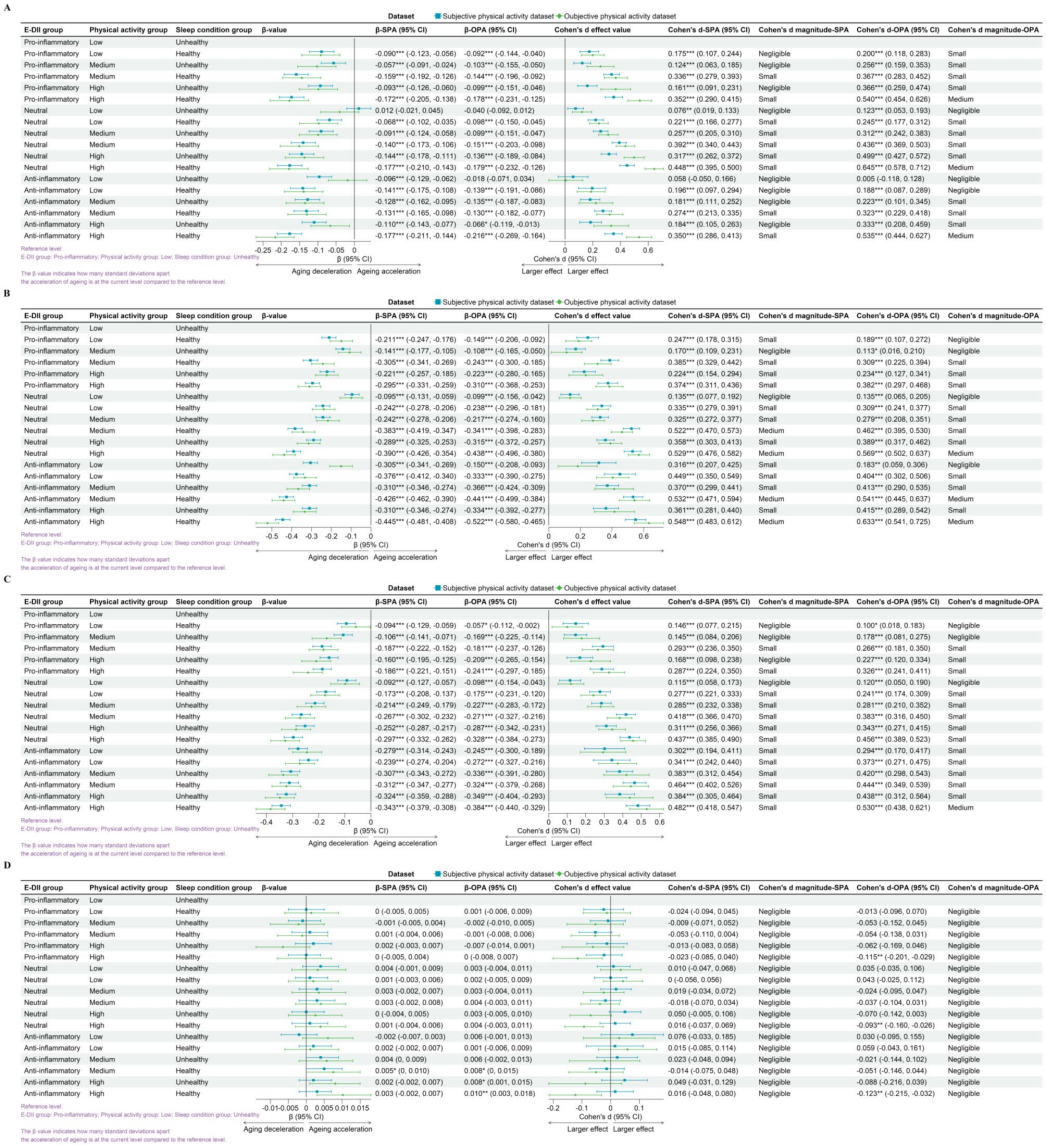
The relationship between lifestyle patterns and the acceleration of biological aging. The figure includes homeostatic dysregulation (**A**), KDM biological age acceleration (**B**), phenotypic age acceleration (**C**), and leucocyte telomere length (**D**). Reference level: proinflammatory in the E-DII group; low in the physical activity group; unhealthy in the sleep condition group. The β-value indicates how many standard deviations of biological age-accelerated change in that level compared to the reference level. Cohen’s d indicates the estimated effect size Abbreviations: SPA: subjective physical activity measurement data set; OPA: objective physical activity measurement data set; E-DII: energy-adjusted dietary inflammation index.

From Fig. 1, it is evident that compared to the reference level (least favorable lifestyle pattern: pro-inflammatory diet, low physical activity, and unhealthy sleep conditions), any improvement in the dietary inflammation index, physical activity, or sleep conditions is associated with lower biological age (Supplementary Material: Figure S3.3 and Figure S3.4). However, these effects are mostly below or equal a moderate effect size.

In the study examining the impact of lifestyle patterns on HD, we observed similar effects across SPA and OPA. The strongest association was observed in the group with a neutral diet, high physical activity level, and healthy sleep conditions, with Cohen’s d for SPA at 0.448 (95% CI: 0.395, 0.500) and for OPA at 0.645 (95% CI: 0.578, 0.712). In the weighted linear model, after adjusting for covariates, the strongest association was observed in the group with an anti-inflammatory diet, high physical activity level, and healthy sleep conditions, with β for SPA at -0.177 (95% CI: -0.211, -0.144) and for OPA at -0.216 (95% CI: -0.269, -0.164).

Regarding the influence of lifestyle patterns on KDM biological age acceleration, similar effects were found across SPA and OPA. The strongest association was present in the group with an anti-inflammatory diet, high physical activity level, and healthy sleep conditions, with Cohen’s d for SPA at 0.548 (95% CI: 0.483, 0.612) and for OPA at 0.633 (95% CI: 0.541, 0.725). Even after adjusting for covariates, this group maintained the strongest association, with β for SPA at -0.445 (95% CI: -0.481, -0.408) and for OPA at -0.522 (95% CI: -0.580, -0.465).

In the study on the impact of lifestyle patterns on phenotypic age acceleration, we found similar effects across SPA and OPA. The strongest association was again observed in the group with an anti-inflammatory diet, high physical activity level, and healthy sleep conditions, with Cohen’s d for SPA at 0.482 (95% CI: 0.418, 0.547) and for OPA at 0.530 (95% CI: 0.438, 0.621). After covariate adjustment, the strongest association persisted in this group, with β for SPA at -0.343 (95% CI: -0.379, -0.308) and for OPA at -0.384 (95% CI: -0.440, -0.329).

In the research on the effects of lifestyle patterns on telomere length, we noted a divergence in the effects between SPA and OPA. In the SPA, a significant association was only observed after covariate adjustment in the group with an anti-inflammatory diet, moderate physical activity level, and healthy sleep conditions, with β for SPA at 0.005 (95% CI: 0, 0.01). Conversely, in the OPA, the strongest association was found in the group with an anti-inflammatory diet, high physical activity level, and healthy sleep conditions, with Cohen’s d for OPA at -0.123 (95% CI: -0.215, -0.032), and the largest effect persisted after covariate adjustment, with β for OPA at 0.01 (95% CI: 0.003, 0.018).

The association between lifestyle patterns and biological age acceleration exhibits significant differences between gender groups only within certain lifestyle groups, with most groups showing no gender-specific variations (Supplementary Material: Figure S3.5 and Figure S3.6). In different age cohorts, individuals under 60 years of age showed stronger associations between lifestyle patterns and HD compared to those over 60. Conversely, the associations between lifestyle patterns and KDM biological aging and phenotypic aging was more pronounced in the population over 60 than in those under 60 (Supplementary Material: Figure S3.7 and Figure S3.8). Within various chronic disease status groups, individuals with a single chronic condition exhibited stronger associations between lifestyle pattern and HD than other groups; those with multimorbidity showed stronger associations with phenotypic age acceleration.

In summary, our findings suggest that maintaining an anti-inflammatory diet, at least moderate level physical activity, and healthy sleep conditions are associated with a significant small to medium level difference in physiological aging markers in most scenarios.

### Biological age aging and all-cause mortality

An elevation of one standard deviation in KDM biological age acceleration is associated with a 30.8% higher all-cause mortality risk (Table 3, HR-SPA = 1.308, 95% CI: 1.273, 1.419), and a similar pattern is observed with a 29.6% increase (HR-OPA = 1.296, 95% CI: 1.232, 1.364). Relative to the reference level (|KDM-age acceleration| ≤ 1), extreme deviations in KDM biological age acceleration (< -1 or > 1) are linked to HR-SPA values of 0.871 (95% CI: 0.802, 0.946) and 1.334 (95% CI: 1.229, 1.447), and HR-OPA values of 0.748 (95% CI: 0.646, 0.866) and 1.213 (95% CI: 1.048, 1.404), respectively.

Similarly, a one standard deviation increase in phenotypic age acceleration is associated with a 33.1% heightened risk of mortality (HR-SPA = 1.331, 95% CI: 1.299, 1.364), and a 28.3% increase (HR-OPA = 1.283, 95% CI: 1.225, 1.344). Against the reference level (|Phenoage acceleration| ≤ 1), participants with Phenoage acceleration < -1 and > 1 exhibit HR-SPA values of 0.867 (95% CI: 0.799, 0.940) and 1.359 (95% CI: 1.256, 1.469), and HR-OPA values of 0.807 (95% CI: 0.696, 0.937) and 1.238 (95% CI: 1.071, 1.430), respectively.

An increase of one standard deviation in telomere length is associated with a 5.4% lower mortality risk (HR-SPA = 0.946, 95% CI: 0.919, 0.972) and a 9.5% decrease (HR-OPA = 0.905, 95% CI: 0.859, 0.953). Compared to the reference level (|Telomere length| ≤ 1), significant telomere length variations (< -1 or > 1) correspond to HR-SPA values of 1.251 (95% CI: 1.168, 1.340) and 0.861 (95% CI: 0.790, 0.939), and HR-OPA values of 1.226 (95% CI: 1.072, 1.402) and 0.837 (95% CI: 0.716, 0.980), respectively.

Kaplan-Meier survival curves substantiate that diminished biological age acceleration or elongated telomere lengths are associated with a reduced all-cause mortality risk (Supplementary Material: Figures S3.11 and S3.12). A non-linear relationship is evident between biological aging acceleration, telomere length, and mortality risk (Supplementary Material: Figure S3.13).

In sum, biological age acceleration and telomere length exhibit significant positive and negative correlations with all-cause mortality. Within the UK Biobank cohort, biological age acceleration emerges as a potent predictor of mortality.

### Optimal lifestyle for reducing all-cause mortality

**Fig. 2:**
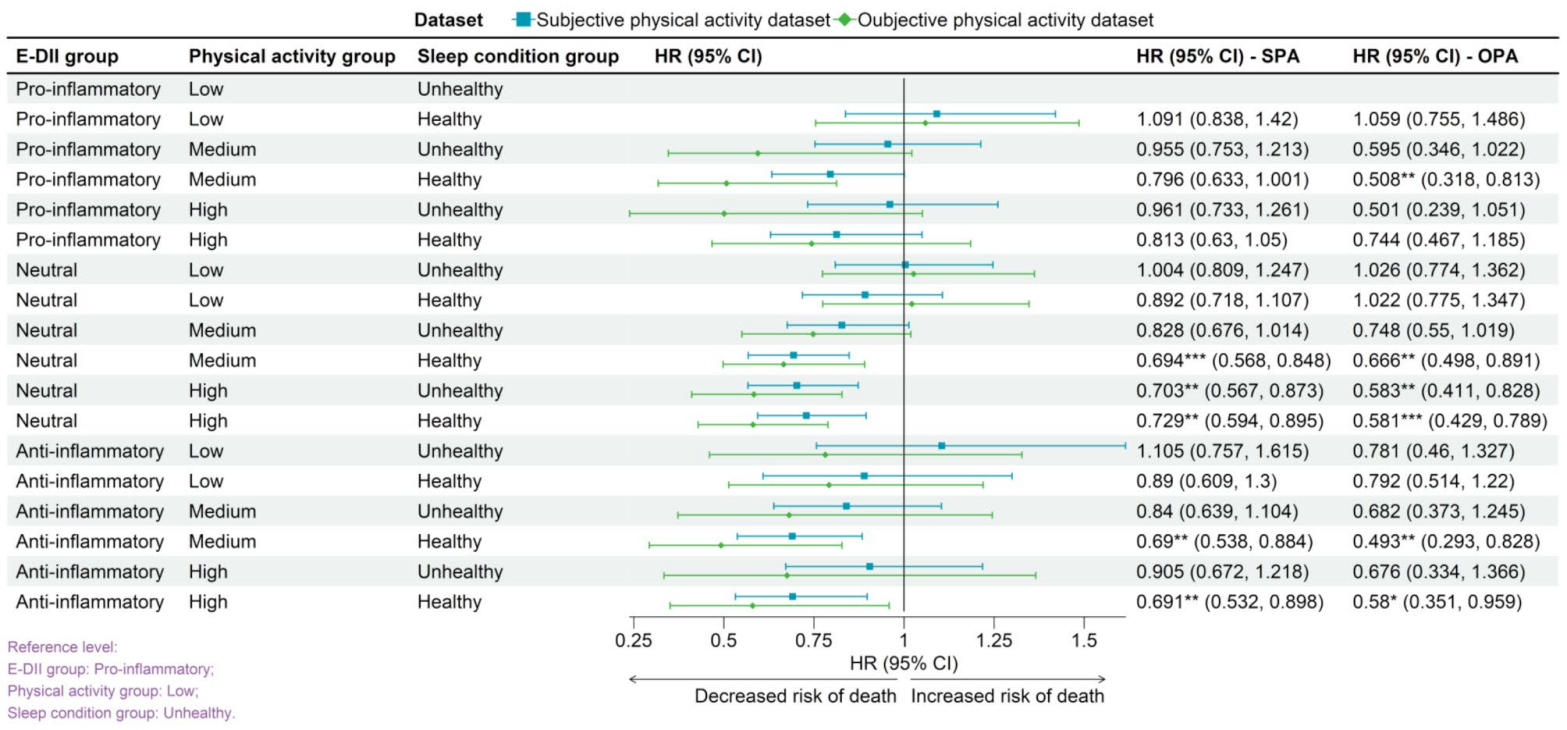
The relationship between lifestyle and all-cause mortality. Reference level: proinflammatory in the E-DII group; low in the physical activity group; unhealthy in the sleep condition group. ^*^P < 0.05, ^**^P < 0.01, ^***^P < 0.001 Abbreviations: SPA: subjective physical activity measurement data set; OPA: objective physical activity measurement data set; E-DII: energy-adjusted dietary inflammation index.

Figure 2 demonstrates that the lifestyle pattern combining an anti-inflammatory diet with moderate physical activity and healthy sleep is associated with the lowest mortality risk (HR-SPA: 0.690, 95% CI: 0.538, 0.884; HR-OPA: 0.493, 95% CI: 0.293, 0.828).

The pro-inflammatory diet group was associated with higher mortality risk compared to those consuming neutral or anti-inflammatory diets. This pattern was mirrored in the associations between physical activity levels, sleep quality, and mortality risk, with higher risks observed in the low physical activity and unhealthy sleep cohorts (Supplementary Material: Figures S3.14 and S3.15, Table S3.1). Additionally, there were linear or non-linear relationships between E-DII, physical activity level, sleep quality score, and the risk of all-cause mortality (Supplementary Material: Figure S3.16).

Disparities in lifestyle associations on mortality risk were evident between genders. While both sexes showed lower mortality risk with moderate physical activity and healthy sleep, women showed a more pronounced association between lower mortality risk and the combination of an anti-inflammatory diet with moderate activity (Supplementary Material: Figures S3.17 - S3.18).

For individuals under 60, a high-activity, healthy-sleep, anti-inflammatory diet lifestyle was associated with significantly lower mortality risk. In contrast, for those aged 60 and above, the association between these lifestyle factors and mortality risk was less pronounced (Supplementary Material: Figures S3.19 - S3.20).

In the absence of chronic diseases, high levels of physical activity and healthy sleep conditions were significantly associated with a lower risk of all-cause mortality (HR approximately 0.769–0.944). For individuals with chronic conditions, an anti-inflammatory diet, particularly when paired with moderate physical activity, was associated with additional lower mortality risk (HR approximately 0.781–0.909). Those with multiple diseases showed lower mortality risk associated with an anti-inflammatory diet across all physical activity levels, especially moderate (HR-SPA = 0.724, 95% CI: 0.547, 0.96) (Supplementary Material: Figures S3.21 - S3.22).

## Discussion

In-depth analysis of large-scale cohort data in this study has revealed associations between lifestyle patterns and the acceleration of biological aging and all-cause mortality. Our findings underscore the relationship between anti-inflammatory diets, high levels of physical activity, and healthy sleep conditions with slower biological aging and lower risk of all-cause mortality.

Our findings regarding the relationship between lifestyle factors and biological aging are consistent with several previous studies, suggesting that healthy lifestyle choices are associated with positive long-term health outcomes [36–39]. For instance, Wang et al. (2023) reported a similar association between higher DII and increased biological age in a cross-sectional study [37]. Our results concerning physical activity align with those of Zhu et al. (2023) [40], who found that moderate to high levels of exercise were associated with reduced biological age across multiple indicators. However, our study extends these findings by examining the combined effects of diet, physical activity, and sleep patterns, aspects that have rarely been comprehensively addressed in previous research. We also observed stronger associations among younger participants and females, adding nuance to the existing literature on lifestyle and aging. Our results concerning the relationship between lifestyle factors and all-cause mortality are largely consistent with the large-scale study by Lu et al. (2024) [41]. However, we observed a stronger protective effect of a comprehensive healthy lifestyle compared to some earlier reports [42]. This discrepancy may be attributed to our use of more detailed lifestyle assessments.

Our data reveal that increases in KDM biological age acceleration and phenotypic age acceleration are significantly associated with an increase in all-cause mortality, highlighting the potential of biological age acceleration as a predictive marker for mortality risk [43]. Furthermore, we observed a negative correlation between telomere length and all-cause mortality, aligning with the existing literature that associates telomere maintenance with extended healthspan [44]. Biological age acceleration may reflect a multitude of biological processes at the cellular level, such as oxidative stress and diminished DNA damage repair capacity [28]. The reduction in telomere length is considered a marker of cellular senescence and tissue function decline [29], while our study finds that increased telomere length correlates with reduced all-cause mortality, suggesting that telomere maintenance plays a key role in prolonging a healthy lifespan.

Healthy lifestyle patterns not only show small to moderate associations with slower biological aging and lower risk of all-cause mortality but also exhibit stable long-term relationships over time, as lifestyle patterns tend to remain consistent with few significant changes [45]. Key components of lifestyle patterns—diet, physical activity, and sleep—are associated with healthspan and mortality risk through various pathways and may also show different relationships with health outcomes in populations with distinct characteristics through diverse combination patterns. An anti-inflammatory diet is associated with slower biological aging, which may be related to lower chronic inflammation and better metabolic health [46]. Increased physical activity is associated with enhanced cardiovascular function and improved efficacy of the immune system [47]. Good sleep quality is associated with endocrine system balance and may be related to the aging process through its relationship with hormone levels [48].

Our analysis has revealed gender and age differences in the associations between lifestyle patterns and biological aging markers. The relationship between lifestyle and biological age acceleration showed significant inter-gender differences in certain lifestyle groups, with most groups exhibiting no gender disparity. Both men and women showed the strongest associations with HD and KDM biological age acceleration, as well as phenotypic age acceleration, in groups adhering to an anti-inflammatory diet, high physical activity levels, and healthy sleep conditions. The differential relationships between lifestyle patterns and HD, KDM biological age, and phenotypic age across age groups highlight the complexity of the aging process. For individuals under 60, lifestyle shows a stronger association with HD, possibly reflecting greater physiological adaptability [49]. Conversely, for those over 60, lifestyle shows stronger associations with KDM biological age and phenotypic age, potentially reflecting the cumulative relationship of long-term lifestyle choices with biological aging and mortality risk [50]. These findings emphasize the importance of health management approaches tailored to different age segments and suggest the potential value of targeted lifestyle modifications in relation to age-related decline. Each biological age measurement method employs distinct algorithms, capturing various aspects from homeostasis to mortality risk, providing a nuanced understanding of how lifestyle patterns shape aging trajectories [28].

While all populations show associations between a healthy lifestyle and lower mortality risk, the relationship between a combination of an anti-inflammatory diet and moderate physical activity and reduced all-cause mortality risk appears to be stronger in women. Additionally, the association between lifestyle factors and reduced all-case mortality risk is stronger in individuals under 60, suggesting potential benefits of early lifestyle modifications. Studies suggest that women may show a stronger association between the combination of an anti-inflammatory diet and physical activity and health outcomes, possibly related to hormonal level differences [51]. Moreover, the biological plasticity of younger individuals may be associated with stronger relationships between lifestyle factors and health outcomes [52].

In individuals with chronic diseases, an anti-inflammatory diet and moderate physical activity are associated with both better protection against mortality and improvements in chronic disease symptoms and overall health. Personalized health strategies, such as individually oriented lifestyle approaches, may be particularly relevant for these individuals, as they consider unique health needs and lifestyle preferences [53]. This comprehensive approach emphasizes collaboration between physicians and patients, as well as active patient participation in their health management, thereby enhancing adherence to treatment plans and overall effectiveness.

In our study, we observe that an anti-inflammatory diet, regular physical activity, and healthy sleep patterns are significantly associated with slower aging and lower all-cause mortality risk. The UK government’s “Eat Well” guide underscores the importance of a balanced diet, advocating for a diverse intake of foods to ensure essential nutrients are met while limiting sugar, salt, and saturated fats. This aligns with the principles of an anti-inflammatory diet, which encourages the selection of foods rich in antioxidants and anti-inflammatory components, such as dark vegetables, whole grains, and Omega-3-rich fish [54].

In addition, the World Health Organization’s ‘Guidelines on Physical Activity and Sedentary Behavior’ recommend that adults should engage in at least 150 to 300 min of moderate-intensity aerobic physical activities, or 75 to 150 min of vigorous-intensity aerobic physical activities per week, along with muscle-strengthening activities. In summary, the minimum level of overall physical activity per week is set at 600 MET-minutes [55]. These guidelines offer a framework to assist policymakers in incorporating more public green spaces and fitness facilities into urban planning, thereby providing the public with convenient exercise environments. Although our research indicates that only high levels (≥ 3000 MET-minutes/week) of physical activity show the strongest association with slower biological aging, this suggests that while the physical activity level recommendations proposed by the WHO are associated with slower biological aging, the strongest relationship may be observed at higher levels of physical activity.

To increase public awareness of the associations between healthy sleep and positive health outcomes, education and media advocacy could be utilized. Through social media, public lectures, and school educational programs, knowledge of sleep hygiene can be disseminated, emphasizing the benefits of regular sleep for physical and mental health.

In summary, by combining the “Eat Well” guide and the recommendations of the World Health Organization [55], we can offer the public a comprehensive framework for a healthy lifestyle. Policymakers could consider these guidelines when formulating environmental and social policies that encourage healthy eating, physical activity, and good sleep habits, which are associated with better public health, slower aging processes, and lower risk of all-cause mortality.

Although our study provides significant insights into the associations between lifestyle patterns and biological age acceleration and all-cause mortality, it also has limitations. Firstly, our study is observational, thus causality cannot be established. Secondly, the assessment of lifestyle patterns, such as diet and sleep, relies on self-reporting, which may introduce bias. Future research should consider using objective methods of lifestyle assessment and validating our findings across different populations.

Third, a key limitation of our study involves the interpretation of associations between biological aging markers and all-cause mortality. Several markers used, including KDM biological age and phenotypic age, were originally developed using mortality data and chronological age as reference points. This inherent relationship could lead to circularity in our findings, potentially overestimating the strength of observed associations. Despite controlling for chronological age, its strong correlation with mortality risk may still confound our results. While these analyses provide value by validating marker performance in our cohort and offering a baseline for lifestyle factor analyses, causal inferences are limited. Future studies might benefit from using biological aging markers developed independently of mortality data.

## Conclusion

Our study confirms that an anti-inflammatory diet, physical activity, and healthy sleep patterns are crucial in slowing biological aging and reducing mortality risk. These lifestyle factors are positively associated with longevity, particularly in individuals under 60 or female. Tailored interventions based on these findings could significantly enhance public health outcomes. Future research should focus on objective lifestyle assessments to further validate these associations.

## Electronic supplementary material

Below is the link to the electronic supplementary material.

Supplementary Material 1

## Data Availability

UKB data are available in a public, open access repository. This research has been conducted using the UK Biobank Resource under Application Number 116198. The UK Biobank data are available on application to the UK Biobank (www.ukbiobank.ac.uk/).

## Abbreviations

CI: Confidence intervals
E-DII: Energy-adjusted Dietary Inflammatory Index
HD: Homeostatic Dysregulation
HRs: Hazard ratios
KDM: Klemera-Doubal Method
NHANES: National Health and Nutrition Examination Survey
OPA: Objective physical activity assessment dataset
SPA: Subjective physical activity assessment dataset
TPA: Total physical activity
