# Supplementary material for: Optimal lifestyle patterns for delaying ageing and reducing all-cause mortality: insights from the UK Biobank
Source: Eur Rev Aging Phys Act. 2024 Oct 5;21:27. doi: 10.1186/s11556-024-00362-7 (PMC11456244; doi:10.1186/s11556-024-00362-7)
Supplement: Supplementary file 1 — Supplementary Material 1 [file 11556_2024_362_MOESM1_ESM.docx]

**Optimal Lifestyle Patterns for Delaying Ageing and Reducing All-Cause Mortality: Insights from the UK Biobank**

**Contents**

S1.	Research process, data sources and variable definitions.	4

Figure S1.1. Exclusions and sample sizes for analyses.	4

Table S1.1. UK Biobank protocol documents.	5

Table S1.2. Variables involved in the study.	6

Table S1.3. 45 nutrients and foods used to calculate the energy-adjusted dietary inflammatory index.	14

Table S1.4. List of the 36 chronic conditions included within the definition of multimorbidity.	16

Table S1.5. Biomarkers considered in biological age algorithms.	17

S2.	Construction and validation of a model for calculating biological age.	20

Figure S2.1. Comparative graph of KDM, PhenoAge, and HD biological aging measures versus actual age in NHANES IV participants.	20

Figure S2.2. Correlation graph between KDM biological age acceleration, PhenoAge age acceleration, and HD (log) biological aging measurements in NHANES IV, including biological ages calculated using both the study-defined algorithms and the original algorithms by Levine.	21

Table S2.1. The relationship between biological aging measures and mortality in NHANES IV, where the coefficients of biological age are estimated as hazard ratios through Cox proportional hazards regression. During the analysis, the KDM biological age and Levine phenotypic age measurements are differentiated from the chronological age (i.e., value = BA - CA). These differences are then standardized for the analysis sample, aligning the mean (M) to 0 and standard deviation (SD) to 1 for both males and females, allowing the effect size to be represented as the gender-specific increase in biological age per 1 SD unit. The model includes covariates of age cohort and gender.	22

Figure S2.3. Density plots of biomarkers in individuals aged 37–73 years in NHANES III (white) and UK Biobank (pink).	25

Figure S2.4. Comparative graph of KDM, PhenoAge, and HD biological aging measures versus actual age in UK Biobank participants.	26

Figure S2.5. Correlation graph of KDM age, phenotypic age, KDM age residuals, phenotypic age residuals, and HD (log) with chronological age in the UK Biobank.	27

Figure S2.6. Density plots of biological age in individuals in men (pink) and women (white).	28

S3.	Supplementary results.	29

Figure S3.1. Histogram of the number of people with different living patterns in the subjective physical activity dataset.	29

Figure S3.2. Histogram of the number of people with different living patterns in the objective physical activity dataset.	30

Figure S3.3. Box plots of HD (A-C), KDM biological age acceleration (D-F), PhenoAge acceleration (G-I), and leukocyte telomere length (J-L) across different diet, physical activity, and sleep condition groups in the subjective physical activity dataset.	31

Figure S3.4. Box plots of HD (A-C), KDM biological age acceleration (D-F), PhenoAge acceleration (G-I), and leukocyte telomere length (J-L) across different diet, physical activity, and sleep condition groups in the objective physical activity dataset.	32

Figure S3.5. The relationship between lifestyle patterns and the acceleration of biological aging across different gender groups within the subjective physical activity measurement dataset. ^*^*P* < 0.05, ^**^*P* < 0.01, ^***^*P* < 0.001.	33

Figure S3.6. The relationship between lifestyle patterns and the acceleration of biological aging across different gender groups within the objective physical activity measurement dataset. ^*^*P* < 0.05, ^**^*P* < 0.01, ^***^*P* < 0.001.	34

Figure S3.7. The relationship between lifestyle patterns and the acceleration of biological aging across different age groups within the subjective physical activity measurement dataset. Note: Young people are defined as age ≤ 60 years and older people are defined as age > 60 years. ^*^*P* < 0.05, ^**^*P* < 0.01, ^***^*P* < 0.001.	35

Figure S3.8. The relationship between lifestyle patterns and the acceleration of biological aging across different age groups within the objective physical activity measurement dataset. Note: Young people are defined as age ≤ 60 years and older people are defined as age > 60 years. ^*^*P* < 0.05, ^**^*P* < 0.01, ^***^*P* < 0.001.	36

Figure S3.9. The relationship between lifestyle patterns and the acceleration of biological aging across different disease condition groups within the subjective physical activity measurement dataset. *^*^P* < 0.05, *^**^P* < 0.01, *^***^P* < 0.001.	37

Figure S3.10. The relationship between lifestyle patterns and the acceleration of biological aging across different disease condition groups within the objective physical activity measurement dataset. ^*^*P* < 0.05, ^**^*P* < 0.01, ^***^*P* < 0.001.	38

Figure S3.11. Survival curves within the subjective physical activity measurement dataset for different KDM biological age acceleration groups (A-C), different PhenoAge acceleration groups (D-F), and different leukocyte telomere length groups (G-I). Biological age acceleration = biological age - chronological age. The biological age acceleration in the graph is z-normalized.	39

Figure S3.12. Survival curves within the objective physical activity measurement dataset for different KDM biological age acceleration groups (A-C), different PhenoAge acceleration groups (D-F), and different leukocyte telomere length groups (G-I). Biological age acceleration = biological age - chronological age. The biological age acceleration in the graph is z-normalized.	40

Figure S3.13. The relationship between biological aging and all-cause mortality in subjective physical activity measurement data sets (A-D) and objective physical activity measurement data sets (E-H).	41

Figure S3.14. Survival curves within the subjective physical activity measurement dataset for different dietary groups (A-C), physical activity groups (D-F), and sleep condition groups (G-I).	42

Figure S3.15. Survival curves within the objective physical activity measurement dataset for different dietary groups (A-C), physical activity groups (D-F), and sleep condition groups (G-I).	43

Figure S3.16. The relationship between lifestyle and all-cause mortality in subjective physical activity measurement data sets (A-C) and objective physical activity measurement data sets (D-F).	44

Table S 3.1. Associations between Energy-Adjusted Dietary Inflammatory Index, Physical Activity, and Sleep Quality with All-Cause Mortality in the UK Biobank Subjective (n = 105705) and Objective (n = 42006) Physical Activity Measurement Datasets.	45

Figure S3.17. The relationship between lifestyle and all-cause mortality across different gender groups within the subjective physical activity measurement dataset. ^*^*P* < 0.05, ^**^*P* < 0.01, ^***^*P* < 0.001.	48

Figure S3.18. The relationship between lifestyle and all-cause mortality across different gender groups within the objective physical activity measurement dataset. ^*^*P* < 0.05, ^**^*P* < 0.01, ^***^*P* < 0.001.	49

Figure S3.19. The relationship between lifestyle and all-cause mortality across different age groups within the subjective physical activity measurement dataset. ^*^*P* < 0.05, ^**^*P* < 0.01, ^***^*P* < 0.001.	50

Figure S3.20. The relationship between lifestyle and all-cause mortality across different age groups within the objective physical activity measurement dataset. ^*^*P* < 0.05, ^**^*P* < 0.01, ^***^*P* < 0.001.	51

Figure S3.21. The relationship between lifestyle and all-cause mortality across different disease condition groups within the subjective physical activity measurement dataset. ^*^*P* < 0.05, ^**^*P* < 0.01, ^***^*P* < 0.001.	52

Figure S3.22. The relationship between lifestyle and all-cause mortality across different disease condition groups within the objective physical activity measurement dataset. ^*^*P* < 0.05, ^**^*P* < 0.01, ^***^*P* < 0.001.	53

S4.	Supplementary method 1: energy-adjusted dietary inflammatory index assessment methods.	54

S5.	Supplementary method 2: physical activity assessment methods.	54

S6.	Supplementary method 3: biological age calculation methods.	55

Reference	56

# S1. Research process, data sources and variable definitions.

Figure S1.1. Exclusions and sample sizes for analyses.

Table S1.1. UK Biobank protocol documents.

| **Document** | **Link** |
| --- | --- |
| Invitation to participate | <https://biobank.ctsu.ox.ac.uk/crystal/refer.cgi?id=100253> |
| Touchscreen questionnaire information | <https://biobank.ndph.ox.ac.uk/showcase/refer.cgi?id=113241> |
| Online 24-hour dietary recall questionnaire | <https://biobank.ndph.ox.ac.uk/showcase/refer.cgi?id=118240> |
| Accelerometer: Participant instructions | <https://biobank.ndph.ox.ac.uk/showcase/refer.cgi?id=141141> |
| Accelerometer: Collection and processing | <https://biobank.ndph.ox.ac.uk/showcase/refer.cgi?id=131600> |
| Death registry linkage | <https://biobank.ctsu.ox.ac.uk/crystal/refer.cgi?id=115559> |
| Assessment centre: Consent | <https://biobank.ndph.ox.ac.uk/showcase/refer.cgi?id=100230> |
| Assessment centre: Reception | <https://biobank.ndph.ox.ac.uk/showcase/ukb/docs/Reception.pdf> |

Table S1.2. Variables involved in the study.

| **Variable** | **Collection method** | **Field ID** | **Description** | **Link** | **Time of assessment** | **Total number of participants** |
| --- | --- | --- | --- | --- | --- | --- |
| Age | Registry | 21003 | Participant age at assessment centre visit (years). Obtained from NHS Primary Care Trust registries and amended by participants at assessment centre visit if required. | <https://biobank.ndph.ox.ac.uk/showcase/field.cgi?id=21003> | Measured at baseline assessment (2006-2010). | 502,166 |
| Sex | Registry | 31 | Obtained from NHS Primary Care Trust registries and amended by participants at assessment centre visit if required. | <https://biobank.ndph.ox.ac.uk/showcase/field.cgi?id=31> | Measured at baseline assessment (2006-2010). | 502,166 |
| Ethnic background | Assessment centre visit: Questionnaire | 21000 | Ethnic group (white, mixed, Asian/Asian British, black/black British, Chinese, other, prefer not to answer) and ethnic background (sub-categories within each ethnic group). | <https://biobank.ndph.ox.ac.uk/showcase/field.cgi?id=21000> | Measured at baseline assessment (2006-2010). | 501,285 |
| Objective physical activity measurement | Accelerometer | 90012 | Average acceleration of Axivity AX3 device across one week collection | <https://biobank.ndph.ox.ac.uk/showcase/field.cgi?id=90012> | Collected during a 7-day period between June 2013 and January 2016. | 103,623 |
| Subjective Physical Activity Measurement | Assessment centre visit: Touchscreen | 100054 | This category contains data from the touchscreen questionnaire on the reported type and duration of physical activity (including walking, DIY, moderate and vigorous physical activity, strenuous sports, etc). This category also includes data on time spent engaging in sedentary activity (driving, watching TV, computer use). | <https://biobank.ndph.ox.ac.uk/showcase/label.cgi?id=100054> | Measured at baseline assessment (2006-2010). | 394,310 |
| Diet by 24-hour recall | Online follow-up | 100090 | This category contains data from a dietary questionnaire, based on a 24-hour dietary recall of the previous day. This questionnaire was added to the Assessment visit towards the end of the recruitment phase. After the close of recruitment 4 additional questionnaire rounds were conducted online, with invitations being emailed to participants at 3-4 monthly intervals. | <https://biobank.ndph.ox.ac.uk/showcase/label.cgi?id=100090> | Assessed through 24-hour recalls collected between April 2009 and June 2012. | 210,856 |
| Current employment status | Assessment centre visit: Questionnaire | 6142 | Paid employment, retired, home/family caretaker, unable to work, unemployed, volunteer, student, other | <https://biobank.ndph.ox.ac.uk/showcase/field.cgi?id=6142> | Measured at baseline assessment (2006-2010). | 501,326 |
| Education | Assessment centre visit: Questionnaire | 6138 | 1 College or University degree  2 A levels/AS levels or equivalent  3 O levels/GCSEs or equivalent  4 CSEs or equivalent  5 NVQ or HND or HNC or equivalent  6 Other professional qualifications eg: nursing, teaching  -7 None of the above  -3 Prefer not to answer | <https://biobank.ndph.ox.ac.uk/showcase/field.cgi?id=6138> | Measured at baseline assessment (2006-2010). | 498,745 |
| Townsend deprivation index | Registry | 189 | Scores corresponding to average home ownership, car ownership, household overcrowding, and employment rate of each participant’s postcode. Calculated based on national census data at time of recruitment. | <https://biobank.ndph.ox.ac.uk/showcase/field.cgi?id=189> | Measured at baseline assessment (2006-2010). | 501,540 |
| Smoking status | Assessment centre visit: Questionnaire | 20116 | Never, previous, current, prefer not to answer | <https://biobank.ndph.ox.ac.uk/showcase/field.cgi?id=20116> | Measured at baseline assessment (2006-2010). | 501,306 |
| Alcohol intake frequency | Assessment centre visit: Questionnaire | 1558 | 1 Daily or almost daily  2 Three or four times a week  3 Once or twice a week  4 One to three times a month  5 Special occasions only  6 Never  -3 Prefer not to answer | <https://biobank.ndph.ox.ac.uk/showcase/field.cgi?id=1558> | Measured at baseline assessment (2006-2010). | 501,300 |
| Body mass index (BMI) | Assessment centre visit: Physical | 21001 | BMI = weight / (standing height)^2^ | <https://biobank.ndph.ox.ac.uk/showcase/field.cgi?id=21001> | Measured at baseline assessment (2006-2010). | 499,223 |
| UK Biobank assessment centre | Assessment centre visit: Assessment centre | 54 | The UK Biobank assessment centre at which participant consented. | <https://biobank.ndph.ox.ac.uk/showcase/field.cgi?id=54> | Measured at baseline assessment (2006-2010). | 502,166 |
| Diagnoses - ICD10 | Health-related outcomes | 41270 | This field is a summary of the distinct diagnosis codes a participant has had recorded across all their hospital inpatient records in either the primary or secondary position. Diagnoses are coded according to the International Classification of Disease version 10 (ICD-10). | <https://biobank.ctsu.ox.ac.uk/crystal/field.cgi?id=41270> | - Hospital Episode Statistics for England (HES): 1997 onwards - 31 October 2022 - Scottish Morbidity Record (SMR): 1996 onwards - 31 August 2022 - Patient Episode Database for Wales (PEDW): 1999 onwards - 31 May 2022 | 446,829 |
| Sleep | Assessment centre visit: Questionnaire | 100057 | This category contains data from the touchscreen questionnaire on duration of sleep, and data on napping, dozing, snoring, sleeplessness and chronotype. | <https://biobank.ndph.ox.ac.uk/showcase/label.cgi?id=100057> | Measured at baseline assessment (2006-2010). | 501,310 |
| Z-adjusted T/S log | Genomics | 22192 | The telomere measures were performed in the laboratory of Dr Veryan Codd and Professor Nilesh J. Samani at the University of Leicester, UK. Full details of the methodology used to measure telomere length, including technical adjustments, can be found in Codd, V. et al. | <https://biobank.ndph.ox.ac.uk/showcase/field.cgi?id=22192> | Measured at baseline assessment (2006-2010). | 473,766 |
| Waist circumference | Assessment centre visit: Physical | 48 | Waist circumference | <https://biobank.ndph.ox.ac.uk/showcase/field.cgi?id=48> | Measured at baseline assessment (2006-2010). | 500,107 |
| Forced expiratory volume in 1-second (FEV1) | Assessment centre visit: Physical | 3063 | FEV1 value calculated from blow | <https://biobank.ndph.ox.ac.uk/showcase/field.cgi?id=3063> | Measured at baseline assessment (2006-2010). | 457,217 |
| Hand grip strength (right) | Assessment centre visit: Physical | 47 | Right grip strength. | <https://biobank.ndph.ox.ac.uk/showcase/field.cgi?id=47> | Measured at baseline assessment (2006-2010). | 499,122 |
| Hand grip strength (left) | Assessment centre visit: Physical | 46 | Left grip strength. | <https://biobank.ndph.ox.ac.uk/showcase/field.cgi?id=46> | Measured at baseline assessment (2006-2010). | 499,053 |
| Albumin | Biological samples | 30600 | Measured by BCG analysis on a Beckman Coulter AU5800 | <https://biobank.ndph.ox.ac.uk/showcase/field.cgi?id=30600> | Measured at baseline assessment (2006-2010). | 432,048 |
| Alkaline phosphatase | Biological samples | 30610 | Measured by AMP(IFCC) analysis on a Beckman Coulter AU5800 | <https://biobank.ndph.ox.ac.uk/showcase/field.cgi?id=30610> | Measured at baseline assessment (2006-2010). | 470,543 |
| Blood urea nitrogen (mmol/L) | Biological samples | 30670 | Measured by GLDH, kinetic analysis on a Beckman Coulter AU5800 | <https://biobank.ndph.ox.ac.uk/showcase/field.cgi?id=30670> | Measured at baseline assessment (2006-2010). | 470,222 |
| Creatinine (umol/L) | Biological samples | 30700 | Measured by enzymatic analysis on a Beckman Coulter AU5800 | <https://biobank.ndph.ox.ac.uk/showcase/field.cgi?id=30700> | Measured at baseline assessment (2006-2010). | 470,307 |
| Serum glucose (mmol/L) | Biological samples | 30740 | Measured by hexokinase analysis on a Beckman Coulter AU5800 | <https://biobank.ndph.ox.ac.uk/showcase/field.cgi?id=30740> | Measured at baseline assessment (2006-2010). | 431,553 |
| Total bilirubin (umol/L) | Biological samples | 30840 | Measured by photometric colour analysis on a Beckman Coulter AU5800 | <https://biobank.ndph.ox.ac.uk/showcase/field.cgi?id=30840> | Measured at baseline assessment (2006-2010). | 468,571 |
| Urate (umol/L) | Biological samples | 30880 | Measured by uricase PAP analysis on a Beckman Coulter AU5800 | <https://biobank.ndph.ox.ac.uk/showcase/field.cgi?id=30880> | Measured at baseline assessment (2006-2010). | 469,981 |
| Basophils percent (%) | Biological samples | 30220 | Result of "Basophils Percentage" assay, performed on blood sample, obtained from UK Biobank assessment centre visit. Basophils (Percentage) is calculated as the proportion of basophils in the leukocytes. | <https://biobank.ndph.ox.ac.uk/showcase/field.cgi?id=30220> | Measured at baseline assessment (2006-2010). | 478,171 |
| Eosinophils percent (%) | Biological samples | 30210 | Result of "Eosinophils Percentage" assay, performed on blood sample, obtained from UK Biobank assessment centre visit. Eosinophils (Percentage) is calculated as the proportion of eosinophils in the leukocytes. | <https://biobank.ndph.ox.ac.uk/showcase/field.cgi?id=30210> | Measured at baseline assessment (2006-2010). | 478,171 |
| Lymphocyte percent (%) | Biological samples | 30180 | Result of "Lymphocytes Percentage" assay, performed on blood sample, obtained from UK Biobank assessment centre visit. Lymphocytes (Percentage) is calculated as the proportion of lymphocytes in the leukocytes. | <https://biobank.ndph.ox.ac.uk/showcase/field.cgi?id=30180> | Measured at baseline assessment (2006-2010). | 478,171 |
| Mean cell volume (fL) | Biological samples | 30270 | Result of "Mean Sphered Cells Volume" assay, performed on blood sample, obtained from UK Biobank assessment centre visit. | <https://biobank.ndph.ox.ac.uk/showcase/field.cgi?id=30270> | Measured at baseline assessment (2006-2010). | 470,726 |
| Monocyte percent (%) | Biological samples | 30190 | Result of "Monocytes Percentage" assay, performed on blood sample, obtained from UK Biobank assessment centre visit. Monocytes (percentage) is calculated as the proportion of monocytes in the leukocytes. | <https://biobank.ndph.ox.ac.uk/showcase/field.cgi?id=30190> | Measured at baseline assessment (2006-2010). | 478,171 |
| Segmented neutrophils percent (%) | Biological samples | 30200 | Result of "Neutrophils Percentage" assay, performed on blood sample, obtained from UK Biobank assessment centre visit. Neutrophils (Percentage) is calculated as the proportion of neutrophils in the leukocytes. | <https://biobank.ndph.ox.ac.uk/showcase/field.cgi?id=30200> | Measured at baseline assessment (2006-2010). | 478,171 |
| Red blood cell count (10^12 cells/L) | Biological samples | 30010 | Result of "Red Blood Cell Count" assay, performed on blood sample, obtained from UK Biobank assessment centre visit. Red blood cells is the number of erythrocytes. Analyser operating range was 0 to 2x10^13 cells/Litre. Typical reference range is 3.9x10^12 to 5.5x10^12 cells/Litre. | <https://biobank.ndph.ox.ac.uk/showcase/field.cgi?id=30010> | Measured at baseline assessment (2006-2010). | 479,033 |
| Red cell distribution width (%) | Biological samples | 30070 | Result of "Red Distribution Width" assay, performed on blood sample, obtained from UK Biobank assessment centre visit. Red blood cell Distribution Width is the size distribution spread of the erythrocyte population derived from the red blood cell histogram. It is the coefficient of variation (CV) expressed in % of the red blood cell size distribution. | <https://biobank.ndph.ox.ac.uk/showcase/field.cgi?id=30070> | Measured at baseline assessment (2006-2010). | 479,032 |
| White blood cell count (10^9 cells/L) | Biological samples | 30000 | Result of "White Blood Cell Count" assay, performed on blood sample, obtained from UK Biobank assessment centre visit. White blood count is the number of leukocytes. Analyser operating range was 0 to 9x10^11 cells/Litre. Typical reference range is 3.5x10^9 to 9.6x10^9 cells/Litre. | <https://biobank.ndph.ox.ac.uk/showcase/field.cgi?id=30000> | Measured at baseline assessment (2006-2010). | 479,028 |
| C-reactive protein (mg/L) | Biological samples | 30710 | Measured by immunoturbidimetric - high sensitivity analysis on a Beckman Coulter AU5800 | <https://biobank.ndph.ox.ac.uk/showcase/field.cgi?id=30710> | Measured at baseline assessment (2006-2010). | 469,541 |
| Cystatin C (mg/L) | Biological samples | 30720 | Measured by latex enhanced immunoturbidimetric analysis on a Siemens ADVIA 1800 | <https://biobank.ndph.ox.ac.uk/showcase/field.cgi?id=30720> | Measured at baseline assessment (2006-2010). | 470,501 |
| Diastolic blood pressure (mm Hg) | Assessment centre visit: Physical | 4079 | Blood pressure, automated reading, diastolic. Two measures of blood pressure were taken a few moments apart.  Range returned by the Omron device is is 0-255 | <https://biobank.ndph.ox.ac.uk/showcase/field.cgi?id=4079> | Measured at baseline assessment (2006-2010). | 475,975 |
| Systolic blood pressure (mm Hg) | Assessment centre visit: Physical | 4080 | Blood pressure, automated reading, systolic. Two measures of blood pressure were taken a few moments apart.  Range returned by the Omron device is is 0-255 | <https://biobank.ndph.ox.ac.uk/showcase/field.cgi?id=4080> | Measured at baseline assessment (2006-2010). | 475,970 |
| Pulse rate | Assessment centre visit: Physical | 102 | This is the pulse rate measured during the automated blood pressure readings. | <https://biobank.ndph.ox.ac.uk/showcase/field.cgi?id=102> | Measured at baseline assessment (2006-2010). | 475,975 |
| Gamma glutamyl transferase (U/L) | Biological samples | 30730 | Measured by IFCC analysis on a Beckman Coulter AU5800 | <https://biobank.ndph.ox.ac.uk/showcase/field.cgi?id=30730> | Measured at baseline assessment (2006-2010). | 470,288 |
| Glycohemoglobin (mmol/mol) | Biological samples | 30750 | Measured by HPLC analysis on a Bio-Rad VARIANT II Turbo | <https://biobank.ndph.ox.ac.uk/showcase/field.cgi?id=30750> | Measured at baseline assessment (2006-2010). | 467,601 |
| HDL cholesterol (mmol/L) | Biological samples | 30760 | Measured by enzyme immunoinhibition analysis on a Beckman Coulter AU5800 | <https://biobank.ndph.ox.ac.uk/showcase/field.cgi?id=30760> | Measured at baseline assessment (2006-2010). | 431,844 |
| LDL direct (mmol/L) | Biological samples | 30780 | Measured by enzymatic protective selection analysis on a Beckman Coulter AU5800 | <https://biobank.ndph.ox.ac.uk/showcase/field.cgi?id=30780> | Measured at baseline assessment (2006-2010). | 469,693 |
| Triglyceride (mmol/L) | Biological samples | 30870 | Measured by GPO-POD analysis on a Beckman Coulter AU5800 | <https://biobank.ndph.ox.ac.uk/showcase/field.cgi?id=30870> | Measured at baseline assessment (2006-2010). | 470,160 |
| Total cholesterol (mmol/L) | Biological samples | 30690 | Measured by CHO-POD analysis on a Beckman Coulter AU5800 | <https://biobank.ndph.ox.ac.uk/showcase/field.cgi?id=30690> | Measured at baseline assessment (2006-2010). | 470,530 |

Table S1.3. 45 nutrients and foods used to calculate the energy-adjusted dietary inflammatory index.

| ID | Name (Units) | Inflammatory effect score (IES) | Global daily mean intake (Units/day) | SD |
| --- | --- | --- | --- | --- |
| 26030 | Alcohol (g) | -0.278 | 13.98 | 3.72 |
| 26021 | Vitamin B12 (μg) | 0.106 | 5.15 | 2.7 |
| 26020 | Vitamin B6 (mg) | -0.365 | 1.47 | 0.74 |
| 26039 | B-Carotene (μg) | -0.584 | 3718 | 1720 |
|  | Caffeine (g) | -0.11 | 8.05 | 6.67 |
| 26013 | Carbohydrate (g) | 0.097 | 272.2 | 40 |
| 26037 | Cholesterol (mg) | 0.11 | 279.4 | 51.2 |
| 26002 | Energy (kcal) | 0.18 | 2056 | 338 |
|  | Eugenol (mg) | -0.14 | 0.01 | 0.08 |
| 26008 | Total fat (g) | 0.298 | 71.4 | 19.4 |
| 26017 | Fibre (g) | -0.663 | 18.8 | 4.9 |
| 26022 | Folic acid (μg) | -0.19 | 273 | 70.7 |
|  | Garlic (g) | -0.412 | 4.35 | 2.9 |
|  | Ginger (g) | -0.453 | 59 | 63.2 |
| 26019 | Fe (mg) | 0.032 | 13.35 | 3.71 |
| 26025 | Mg (mg) | -0.484 | 310.1 | 139.4 |
| 26032 | MUFA(g) | -0.009 | 27 | 6.1 |
| 26054 | Niacin (mg) | -0.246 | 25.9 | 11.72 |
| 26015 | n-3 Fatty acids (g) | -0.436 | 1.06 | 1.06 |
| 26016 | n-6 Fatty acids (g) | -0.159 | 10.8 | 7.5 |
|  | Onion (g) | -0.301 | 35.9 | 18.4 |
| 26005 | Protein (g) | 0.021 | 79.4 | 13.9 |
|  | PUFA (g) | -0.337 | 13.88 | 3.76 |
| 26035 | Riboflavin (mg) | -0.068 | 1.7 | 0.79 |
|  | Saffron (g) | -0.14 | 0.37 | 1.78 |
| 26014 | Saturated fat (g) | 0.373 | 28.6 | 8 |
| 26058 | Se (ug) | -0.191 | 67 | 25.1 |
| 26034 | Thiamin (mg) | -0.098 | 1.7 | 0.66 |
| 26155 | Trans fat (g) | 0.229 | 3.15 | 3.75 |
|  | Tumeric (mg) | -0.785 | 533.6 | 754.3 |
| 26061 | Vitamin A (RE) | -0.401 | 983.9 | 518.6 |
| 26023 | Vitamin C (mg) | -0.424 | 118.2 | 43.46 |
| 26029 | Vitamin D (ug) | -0.446 | 6.26 | 2.21 |
| 26028 | Vitamin E (mg) | -0.419 | 8.73 | 1.49 |
| 26033 | Zn (mg) | -0.313 | 9.84 | 2.19 |
|  | Green/black tea (g) | -0.536 | 1.69 | 1.53 |
|  | Flavan-3-ol (mg) | -0.415 | 95.8 | 85.9 |
|  | Flavones (mg) | -0.616 | 1.55 | 0.07 |
|  | Flavonols (mg) | -0.467 | 17.7 | 6.79 |
|  | Flavonones (mg) | -0.25 | 11.7 | 3.82 |
|  | Anthocyanidins (mg) | -0.131 | 18.05 | 21.14 |
|  | Isoflavones (mg) | -0.593 | 1.2 | 0.2 |
|  | Pepper (g) | -0.131 | 10 | 7.07 |
|  | Thyme/oregano (mg) | -0.102 | 0.33 | 0.99 |
|  | Rosemary (mg) | -0.013 | 1 | 15 |

Table S1.4. List of the 36 chronic conditions included within the definition of multimorbidity.

| Name of the disease | ICD-10 code |
| --- | --- |
| Anemia | D50, D51, D52, D53, D55, D56, D57, D58, D59, D60, D61, D63, D64 |
| Angina | I20 |
| Anxiety or panic attacks | F40, F41, F42, F43 |
| Asthma | J45 |
| Atrial fibrillation | I48 |
| Bronchiectasis | J47 |
| Cancer | C00-C97 |
| Chronic kidney disease | N18 |
| Chronic obstructive pulmonary disease (COPD) | J44 |
| Chronic sinusitis | J32 |
| Cirrhosis | K70.3, K74, K86.8 |
| Dementia | F00, F01, F02, F03, F04, F05, F06, F07, F09 |
| Depression | F31, F32, F33, F34, F38 |
| Diabetes | E10-E14 |
| Eczema or dermatitis | L20-L30 |
| Epilepsy | G40 |
| Glaucoma | H40 |
| Heart failure | I50 |
| Hepatitis | B15, B16, B17, B18, B19, K75.9, K73, K70.1 |
| Hypertension | I10-I15 |
| Inflammatory bowel disease | K50, K51 |
| Irritable bowel syndrome | K58 |
| Meningitis | G00-G03 |
| Migraine | G43 |
| Multiple sclerosis | G35 |
| Myocardial infarction | I21-I22 |
| Osteoporosis | M80-M82 |
| Parkinson’s disease | G20-G22 |
| Peripheral vascular disease | I73, I74 |
| Prostate problem | N40-N42 |
| Rheumatoid arthritis | M05, M06 |
| Schizophrenia | F20, F21, , F22, F23, F24, F25, F28, F29 |
| Stroke | I61, I63, I64 |
| Thyroid problem | E00-E07 |
| Tuberculosis | A15-A19 |

Table S1.5. Biomarkers considered in biological age algorithms.

| **Variable Name** | **Description** | **Field ID** | **C/N** | **Available in NHANES** |
| --- | --- | --- | --- | --- |
| **Demographic** | | | | |
| eid | Sample ID |  | Character |  |
| gender | Gender of the sample person | 31 | Numeric | Yes |
| age | Age at baseline |  | Numeric | Yes |
| bmi | Body mass index (kg/m^2^) | 21001 | Numeric | Yes |
| height | Standing height (cm) |  | Numeric | Yes |
| waist | Waist circumference (cm) | 48 | Numeric | Yes |
| weight | Weight (kg) |  | Numeric | Yes |
| fev | Forced expiratory volume in the first 1.0 second (mL) |  | Numeric | Yes |
| fev_1000 | Forced expiratory volume (L) | 3063 | Numeric | Yes |
| grip_r | Grip strength, right hand (kg) | 47 | Numeric | Yes |
| grip_l | Grip strength, left hand (kg) | 46 | Numeric | Yes |
| **Biomarkers** | | | | |
| albumin_gL | Albumin (g/L) | 30600 | Numeric | Yes |
| albumin | Albumin (g/dL) |  | Numeric | Yes |
| alp | Alkaline phosphatase (U/L) | 30610 | Numeric | Yes |
| lnalp | Log (alkaline phosphatase) (U/L) |  | Numeric | Yes |
| bun_mmol | Blood urea nitrogen (mmol/L) | 30670 | Numeric | No |
| bun | Blood urea nitrogen (mg/dL) |  | Numeric | Yes |
| lnbun | Log (blood urea nitrogen) (mg/dL) |  | Numeric | Yes |
| creat | Creatinine (mg/dL) |  | Numeric | Yes |
| creat_umol | Creatinine (umol/L) | 30700 | Numeric | Yes |
| lncreat | Log (creatinine) (mg/dL) |  | Numeric | Yes |
| lncreat_umol | Log (creatinine) (umol/L) |  | Numeric | Yes |
| glucose | Serum glucose (mg/dL) |  | Numeric | Yes |
| glucose_mmol | Serum glucose (mmol/L) | 30740 | Numeric | Yes |
| ttbl_umol | Total bilirubin (umol/L) | 30840 | Numeric | No |
| ttbl | Total bilirubin (mg/dL) |  | Numeric | Yes |
| uap_umol | Urate (umol/L) | 30880 | Numeric | No |
| uap | Uric acid (mg/dL) |  | Numeric | Yes |
| lnuap | Log (uric acid) (mg/dL) |  | Numeric | Yes |
| basopa | Basophils percent (%) | 30220 | Numeric | Yes |
| eosnpa | Eosinophils percent (%) | 30210 | Numeric | Yes |
| lymph | Lymphocyte percent (%) | 30180 | Numeric | Yes |
| mcv | Mean cell volume (fL) | 30270 | Numeric | Yes |
| monopa | Monocyte percent (%) | 30190 | Numeric | Yes |
| neut | Segmented neutrophils percent (%) | 30200 | Numeric | Yes |
| rbc_L | Red blood cell count (10^12 cells/L) | 30010 | Numeric | No |
| rbc | Red blood cell count (million cells/uL) |  | Numeric | Yes |
| rdw | Red cell distribution width (%) | 30070 | Numeric | Yes |
| wbc_L | White blood cell count (10^9 cells/L) | 30000 | Numeric | No |
| wbc | White blood cell count (1000 cells/uL) |  | Numeric | Yes |
| crp_mgL | C-reactive protein (mg/L) | 30710 | Numeric | No |
| crp | C-reactive protein (mg/dL) |  | Numeric | Yes |
| lncrp | Log (c-reactive protein) (mg/dL) |  | Numeric | Yes |
| cyst | Cystatin C (mg/L) | 30720 | Numeric | Yes |
| dbp | Diastolic blood pressure (mm Hg) | 4079 | Numeric | Yes |
| sbp | Systolic blood pressure (mm Hg) | 4080 | Numeric | Yes |
| pulse | Pulse rate | 102 | Numeric | Yes |
| ggt | Gamma glutamyl transferase (U/L) | 30730 | Numeric | Yes |
| hba1c_mmol | Glycohemoglobin (mmol/mol) | 30750 | Numeric | No |
| hba1c | Glycohemoglobin (%) |  | Numeric | Yes |
| lnhba1c | Log (glycohemoglobin) (%) |  | Numeric | Yes |
| hdl_mmol | HDL cholesterol (mmol/L) | 30760 | Numeric | No |
| hdl | HDL (mg/dL) |  | Numeric | Yes |
| ldl_mmol | LDL direct (mmol/L) | 30780 | Numeric | No |
| ldl | LDL (mg/dL) |  | Numeric | Yes |
| trig_mmol | Triglyceride (mmol/L) | 30870 | Numeric | No |
| trig | Triglyceride (mg/dL) |  | Numeric | Yes |
| totchol_mmol | Total cholesterol (mmol/L) | 30690 | Numeric | No |
| totchol | Total cholesterol (mg/dL) |  | Numeric | Yes |

# S2. Construction and validation of a model for calculating biological age.

Figure S2.1. Comparative graph of KDM, PhenoAge, and HD biological aging measures versus actual age in NHANES IV participants.

Abbreviations: HD, homeostatic dysregulation; KDM, Klemera-Doubal method; NHANES, National Health and Nutrition Examination Survey

Figure S2.2. Correlation graph between KDM biological age acceleration, PhenoAge age acceleration, and HD (log) biological aging measurements in NHANES IV, including biological ages calculated using both the study-defined algorithms and the original algorithms by Levine.

Abbreviations: HD, homeostatic dysregulation; KDM, Klemera-Doubal method; NHANES, National Health and Nutrition Examination Survey

Table S2.1. The relationship between biological aging measures and mortality in NHANES IV, where the coefficients of biological age are estimated as hazard ratios through Cox proportional hazards regression. During the analysis, the KDM biological age and Levine phenotypic age measurements are differentiated from the chronological age (i.e., value = BA - CA). These differences are then standardized for the analysis sample, aligning the mean (M) to 0 and standard deviation (SD) to 1 for both males and females, allowing the effect size to be represented as the gender-specific increase in biological age per 1 SD unit. The model includes covariates of age cohort and gender.

|  | **KDM residual** | **PhenoAge residual** | **HD (log)** | **Levine original KDM residual** | **Levine original PhenoAge residual** |
| --- | --- | --- | --- | --- | --- |
| **Hazard Ratio (95% CI)** | | | | | |
| **Full Sample** | | | | | |
| n | 3849 | 3849 | 3849 | 3849 | 3849 |
| BioAge | 1.62 (1.35, 1.96) | 1.81 (1.56, 2.1) | 1.5 (1.19, 1.88) | 1.48 (1.22, 1.79) | 1.73 (1.5, 1.99) |
| **Stratified by Gender** | | | | | |
| **Men** | | | | | |
| n | 1919 | 1919 | 1919 | 1919 | 1919 |
| BioAge | 1.82 (1.45, 2.29) | 1.77 (1.48, 2.11) | 1.53 (1.16, 2.01) | 1.61 (1.28, 2.02) | 1.72 (1.45, 2.03) |
| **Women** | | | | | |
| n | 1930 | 1930 | 1930 | 1930 | 1930 |
| BioAge | 1.33 (0.96, 1.83) | 1.97 (1.49, 2.61) | 1.43 (0.95, 2.17) | 1.27 (0.91, 1.76) | 1.82 (1.41, 2.36) |
| **Stratified by Race** | | | | | |
| **White** | | | | | |
| n | 1810 | 1810 | 1810 | 1810 | 1810 |
| BioAge | 1.58 (1.23, 2.03) | 2 (1.58, 2.52) | 1.5 (1.12, 2.01) | 1.49 (1.15, 1.93) | 1.88 (1.53, 2.32) |
| **Black** | | | | | |
| n | 675 | 675 | 675 | 675 | 675 |
| BioAge | 1.94 (1.35, 2.78) | 1.65 (1.24, 2.19) | 2.83 (1.66, 4.81) | 1.58 (1.1, 2.27) | 1.59 (1.23, 2.06) |
| **Other** | | | | | |
| n | 1364 | 1364 | 1364 | 1364 | 1364 |
| BioAge | 1.57 (0.94, 2.62) | 2.16 (1.27, 3.69) | 0.82 (0.45, 1.5) | 1.63 (0.94, 2.83) | 1.59 (0.99, 2.54) |
| **People Aged 65 and Younger** | | | | | |
| **Aged 65 and Younger** | | | | | |
| n | 3242 | 3242 | 3242 | 3242 | 3242 |
| BioAge | 1.57 (1.21, 2.03) | 1.7 (1.42, 2.05) | 1.33 (0.99, 1.77) | 1.42 (1.09, 1.86) | 1.69 (1.41, 2.02) |

Abbreviations: HD, homeostatic dysregulation; KDM, Klemera-Doubal method; NHANES, National Health and Nutrition Examination Survey

Figure S2.3. Density plots of biomarkers in individuals aged 37–73 years in NHANES III (white) and UK Biobank (pink).

Figure S2.4. Comparative graph of KDM, PhenoAge, and HD biological aging measures versus actual age in UK Biobank participants.

Abbreviations: HD, homeostatic dysregulation; KDM, Klemera-Doubal method.

Figure S2.5. Correlation graph of KDM age, phenotypic age, KDM age residuals, phenotypic age residuals, and HD (log) with chronological age in the UK Biobank.

Abbreviations: HD, homeostatic dysregulation; KDM, Klemera-Doubal method; PhenoAge, phenotype age

Figure S2.6. Density plots of biological age in individuals in men (pink) and women (white).

Abbreviations: HD, homeostatic dysregulation; KDM, Klemera-Doubal method; PhenoAge, phenotype age

# S3. Supplementary results.

Figure S3.1. Histogram of the number of people with different living patterns in the subjective physical activity dataset.

Note: For instance, in ‘000’, the first ‘0’ denotes a pro-inflammatory diet, the second ‘0’ indicates low physical activity, and the third ‘0’ signifies unhealthy sleep quality. Conversely, ‘1’ represents a neutral diet, or moderate physical activity, or healthy sleep quality. Similarly, ‘2’ corresponds to an anti-inflammatory diet, or high physical activity.

Figure S3.2. Histogram of the number of people with different living patterns in the objective physical activity dataset.

Note: For instance, in ‘000’, the first ‘0’ denotes a pro-inflammatory diet, the second ‘0’ indicates low physical activity, and the third ‘0’ signifies unhealthy sleep quality. Conversely, ‘1’ represents a neutral diet, or moderate physical activity, or healthy sleep quality. Similarly, ‘2’ corresponds to an anti-inflammatory diet, or high physical activity.

**S3.1 Supplementary results of aging.**

Figure S3.3. Box plots of HD (A-C), KDM biological age acceleration (D-F), PhenoAge acceleration (G-I), and leukocyte telomere length (J-L) across different diet, physical activity, and sleep condition groups in the subjective physical activity dataset.

Abbreviations: HD, homeostatic dysregulation; KDM, Klemera-Doubal method; PhenoAge, phenotype age

Figure S3.4. Box plots of HD (A-C), KDM biological age acceleration (D-F), PhenoAge acceleration (G-I), and leukocyte telomere length (J-L) across different diet, physical activity, and sleep condition groups in the objective physical activity dataset.

Abbreviations: HD, homeostatic dysregulation; KDM, Klemera-Doubal method; PhenoAge, phenotype age

Figure S3.5. The relationship between lifestyle patterns and the acceleration of biological aging across different gender groups within the subjective physical activity measurement dataset. ^*^*P* < 0.05, ^**^*P* < 0.01, ^***^*P* < 0.001.

Figure S3.6. The relationship between lifestyle patterns and the acceleration of biological aging across different gender groups within the objective physical activity measurement dataset. ^*^*P* < 0.05, ^**^*P* < 0.01, ^***^*P* < 0.001.

Figure S3.7. The relationship between lifestyle patterns and the acceleration of biological aging across different age groups within the subjective physical activity measurement dataset. Note: Young people are defined as age ≤ 60 years and older people are defined as age > 60 years. ^*^*P* < 0.05, ^**^*P* < 0.01, ^***^*P* < 0.001.

Figure S3.8. The relationship between lifestyle patterns and the acceleration of biological aging across different age groups within the objective physical activity measurement dataset. Note: Young people are defined as age ≤ 60 years and older people are defined as age > 60 years. ^*^*P* < 0.05, ^**^*P* < 0.01, ^***^*P* < 0.001.

Figure S3.9. The relationship between lifestyle patterns and the acceleration of biological aging across different disease condition groups within the subjective physical activity measurement dataset. *^*^P* < 0.05, *^**^P* < 0.01, *^***^P* < 0.001.

Figure S3.10. The relationship between lifestyle patterns and the acceleration of biological aging across different disease condition groups within the objective physical activity measurement dataset. ^*^*P* < 0.05, ^**^*P* < 0.01, ^***^*P* < 0.001.

**S3.2 Supplementary results on all-cause mortality.**

Figure S3.11. Survival curves within the subjective physical activity measurement dataset for different KDM biological age acceleration groups (A-C), different PhenoAge acceleration groups (D-F), and different leukocyte telomere length groups (G-I). Biological age acceleration = biological age - chronological age. The biological age acceleration in the graph is z-normalized.

Abbreviations: KDM, Klemera-Doubal method; PhenoAge, phenotype age

Figure S3.12. Survival curves within the objective physical activity measurement dataset for different KDM biological age acceleration groups (A-C), different PhenoAge acceleration groups (D-F), and different leukocyte telomere length groups (G-I). Biological age acceleration = biological age - chronological age. The biological age acceleration in the graph is z-normalized.

Abbreviations: KDM, Klemera-Doubal method; PhenoAge, phenotype age

Figure S3.13. The relationship between biological aging and all-cause mortality in subjective physical activity measurement data sets (A-D) and objective physical activity measurement data sets (E-H).

Abbreviations: KDM, Klemera-Doubal method; HR, Hazard ratio.

Figure S3.14. Survival curves within the subjective physical activity measurement dataset for different dietary groups (A-C), physical activity groups (D-F), and sleep condition groups (G-I).

Abbreviations: E-DII, Energy-adjusted dietary inflammation index; TPA, Total physical activity.

Figure S3.15. Survival curves within the objective physical activity measurement dataset for different dietary groups (A-C), physical activity groups (D-F), and sleep condition groups (G-I).

Abbreviations: E-DII, Energy-adjusted dietary inflammation index; TPA, Total physical activity

Figure S3.16. The relationship between lifestyle and all-cause mortality in subjective physical activity measurement data sets (A-C) and objective physical activity measurement data sets (D-F).

Abbreviations: MET, metabolic equivalents; HR, Hazard ratio.

Table S 3.1. Associations between Energy-Adjusted Dietary Inflammatory Index, Physical Activity, and Sleep Quality with All-Cause Mortality in the UK Biobank Subjective (n = 105705) and Objective (n = 42006) Physical Activity Measurement Datasets.

| Life pattern | HR (95% CI) | | | | | |
| --- | --- | --- | --- | --- | --- | --- |
|  | Subjective physical activity dataset | | | Objective physical activity dataset | | |
|  | All (N = 105705) | Male (N = 49312) | Female (N = 56393) | All (N = 42006) | Male (N = 18291) | Female (N = 23715) |
| E-DII ^a^ |  |  |  |  |  |  |
| Model 1 |  |  |  |  |  |  |
| E-DII, continuous (per standard deviation) | 1.094^***^ (1.065, 1.124) | 1.092^***^ (1.057, 1.128) | 1.089^***^ (1.044, 1.137) | 1.108^***^ (1.054, 1.165) | 1.099^**^ (1.034, 1.170) | 1.108^**^ (1.026, 1.197) |
| E-DII, categorical |  |  |  |  |  |  |
| Pro-inflammatory | 1.000 | 1.000 | 1.000 | 1.000 | 1.000 | 1.000 |
| Neutral | 0.786^***^ (0.731, 0.845) | 0.779^***^ (0.707, 0.859) | 0.797^***^ (0.714, 0.888) | 0.885 (0.765, 1.024) | 0.916 (0.747, 1.123) | 0.859 (0.697, 1.059) |
| Anti-inflammatory | 0.785^***^ (0.705, 0.875) | 0.782^***^ (0.677, 0.904) | 0.789^**^ (0.672, 0.929) | 0.709^**^ (0.564, 0.892) | 0.792 (0.581, 1.79) | 0.625 (0.443, 0.879) |
| Model 2 |  |  |  |  |  |  |
| E-DII, continuous (per standard deviation) | 1.047^**^ (1.018, 1.075) | 1.045^***^ (1.011, 1.079) | 1.044^*^ (1.001, 1.089) | 1.056^*^ (1.004, 1.111) | 1.066^*^ (1.002, 1.135) | 1.036 (0.958, 1.119) |
| E-DII, categorical |  |  |  |  |  |  |
| Pro-inflammatory | 1.000 | 1.000 | 1.000 | 1.000 | 1.000 | 1.000 |
| Neutral | 0.750^*^ (0.567, 0.992) | 0.758^*^ (0.578, 0.995) | 0.845^**^ (0.757, 0.944) | 0.955 (0.825, 1.106) | 1.076 (0.874, 1.324) | 0.903 (0.686, 1.189) |
| Anti-inflammatory | 0.556^*^ (0.335, 0.924) | 0.521^*^ (0.298, 0.912) | 0.838^*^ (0.712, 0.988) | 0.790^*^ (0.628, 0.994) | 0.991 (0.724, 1.355) | 0.595^*^ (0.356, 0.993) |
| Physical activity ^b^ |  |  |  |  |  |  |
| Model 1 |  |  |  |  |  |  |
| Physical activity, continuous (per standard deviation) | 0.945^***^ (0.919, 0.971) | 0.949^**^ (0.916, 0.983) | 0.937^***^ (0.896, 0.979) | 0.641^***^ (0.602, 0.683) | 0.647^***^ (0.596, 0.702) | 0.628^***^ (0.568, 0.693) |
| Physical activity, categorical |  |  |  |  |  |  |
| Low | 1.000 | 1.000 | 1.000 | 1.000 | 1.000 | 1.000 |
| Moderate | 0.727^***^ (0.677, 0.779) | 0.701^***^ (0.642, 0.766) | 0.797^***^ (0.714, 0.888) | 0.596^***^ (0.530, 0.669) | 0.615^***^ (0.529, 0.714) | 0.568^***^ (0.472, 0.685) |
| High | 0.718^***^ (0.664, 0.775) | 0.699^***^ (0.635, 0.770) | 0.789^**^ (0.672, 0.929) | 0.511^***^ (0.446, 0.585) | 0.517^***^ (0.433, 0.618) | 0.501^***^ (0.407, 0.617) |
| Model 2 |  |  |  |  |  |  |
| Physical activity, continuous (per standard deviation) | 0.958^**^ (0.933, 0.985) | 0.962^*^ (0.929, 0.997) | 0.951^*^ (0.910, 0.995) | 0.696^***^ (0.653, 0.743) | 0.704^***^ (0.647, 0.767) | 0.679^***^ (0.612, 0.753) |
| Physical activity, categorical |  |  |  |  |  |  |
| Low | 1.000 | 1.000 | 1.000 | 1.000 | 1.000 | 1.000 |
| Moderate | 0.801^***^ (0.745, 0.859) | 0.774^***^ (0.708, 0.846) | 0.834^**^ (0.740, 0.939) | 0.661^***^ (0.587, 0.744) | 0.672^***^ (0.577, 0.782) | 0.634^***^ (0.524, 0.767) |
| High | 0.787^***^ (0.727, 0.851) | 0.766^***^ (0.694, 0.845) | 0.816^**^ (0.715, 0.930) | 0.592^***^ (0.514, 0.681) | 0.598^***^ (0.497, 0.719) | 0.580^***^ (0.467, 0.722) |
| Sleep conditions ^c^ |  |  |  |  |  |  |
| Model 1 ^d^ |  |  |  |  |  |  |
| Sleep conditions, continuous (per standard deviation) | 0.890^***^ (0.868, 0.913) | 0.908^***^ (0.879, 0.938) | 0.863^***^ (0.828, 0.899) | 0.910^***^ (0.867, 0.956) | 0.946 (0.888, 1.007) | 0.862^***^ (0.799, 0.929) |
| Sleep conditions, categorical |  |  |  |  |  |  |
| Unhealthy | 1.000 | 1.000 | 1.000 | 1.000 | 1.000 | 1.000 |
| Healthy | 0.808^***^ (0.766, 0.852) | 0.840^***^ (0.785, 0.899) | 0.756^***^ (0.693, 0.825) | 0.861^**^ (0.778, 0.953) | 0.936 (0.822, 1.067) | 0.755^***^ (0.642, 0.887) |
| Model 2 ^e^ |  |  |  |  |  |  |
| Sleep conditions, continuous (per standard deviation) | 0.943^*^ (0.892, 0.996) | 0.962^*^ (0.931, 0.995) | 0.893^***^ (0.857, 0.932) | 0.811^**^ (0.695, 0.947) | 0.712^*^ (0.529, 0.956) | 0.925^*^ (0.856, 0.999) |
| Sleep conditions, categorical |  |  |  |  |  |  |
| Unhealthy | 1.000 | 1.000 | 1.000 | 1.000 | 1.000 | 1.000 |
| Healthy | 0.875^***^ (0.829, 0.924) | 0.926^*^ (0.865, 0.992) | 0.829^***^ (0.758, 0.905) | 0.968 (0.874, 1.074) | 1.054 (0.923, 1.203) | 0.841^*^ (0.714, 0.989) |

^*^ *P*<0.05, ^**^ *P*<0.01, ^***^ *P*<0.001.

^a^ Energy-adjusted dietary inflammation index.

^b^ Total physical activity as assessed by the modified international physical activity questionnaire in the subjective physical activity measurement dataset; and the mean overall acceleration measured by the Axivity AX3 wrist-worn triaxial accelerometer (Axivity Ltd., Newcastle, UK) in the objective physical activity measurement dataset.

^c^ Healthy sleep was characterized by five specific criteria: a morning chronotype, 7-8 hours of sleep daily, few or no insomnia symptoms, no snoring, and little to no daytime sleepiness.

^d^ Sex and age were adjusted in the full population, and age was adjusted in the sex subgroups. In models where age had a non-linear effect, the non-linear term for age was adjusted.

^e^ The model was adjusted for age, gender, dietary inflammatory index, physical activity, sleep status, Townsend deprivation index, employment status, education level, ethnicity, smoking status, frequency of alcohol consumption, body mass index, UK Biobank assessment center location, and comorbidities. Non-linear terms for age were adjusted in models where age had a non-linear effect. Additionally, menopausal status was further adjusted in the female subgroup.

Figure S3.17. The relationship between lifestyle and all-cause mortality across different gender groups within the subjective physical activity measurement dataset. ^*^*P* < 0.05, ^**^*P* < 0.01, ^***^*P* < 0.001.

Figure S3.18. The relationship between lifestyle and all-cause mortality across different gender groups within the objective physical activity measurement dataset. ^*^*P* < 0.05, ^**^*P* < 0.01, ^***^*P* < 0.001.

Figure S3.19. The relationship between lifestyle and all-cause mortality across different age groups within the subjective physical activity measurement dataset. ^*^*P* < 0.05, ^**^*P* < 0.01, ^***^*P* < 0.001.

Figure S3.20. The relationship between lifestyle and all-cause mortality across different age groups within the objective physical activity measurement dataset. ^*^*P* < 0.05, ^**^*P* < 0.01, ^***^*P* < 0.001.

Figure S3.21. The relationship between lifestyle and all-cause mortality across different disease condition groups within the subjective physical activity measurement dataset. ^*^*P* < 0.05, ^**^*P* < 0.01, ^***^*P* < 0.001.

Figure S3.22. The relationship between lifestyle and all-cause mortality across different disease condition groups within the objective physical activity measurement dataset. ^*^*P* < 0.05, ^**^*P* < 0.01, ^***^*P* < 0.001.

# S4. Supplementary method 1: energy-adjusted dietary inflammatory index assessment methods.

The Dietary Inflammatory Index (DII), proposed by Shivappa et al. in 2014, encompasses 45 distinct dietary factors (including nutrients and foods) and categorizes them based on their positive (pro-inflammatory) or negative (anti-inflammatory) effects, as determined through extensive literature reviews [1]. In our study, we incorporated 27 food items and nutrients available in the UK Biobank, including Alcohol, Vitamin B12, Vitamin B6, Beta-carotene, Carbohydrate, Cholesterol, Energy, Fat, Englyst fiber, Folate, Iron, Magnesium, Monounsaturated fatty acids, Niacin equivalent, n-3 fatty acids, n-6 fatty acids, Protein, Riboflavin, Saturated fatty acids, Selenium, Thiamin, Trans fatty acids, Vitamin A retinol equivalents, Vitamin C, Vitamin D, and Zinc (Supplementary material: Table S1.3). These intake values were standardized to per 1000 kcal per day, consistent with global average daily intakes, to mitigate the impact of varying energy intake among participants.

The calculation method for the E- DII is as follows: First, we subtracted each nutrient or food item’s value from the corresponding global average daily intake and divided it by the standard deviation to obtain a z-score. Next, we transformed the z-scores into percentile scores, doubling each percentile score and subtracting it from 1 to mitigate data skewness. Finally, we multiplied the resulting value by the relevant food-specific inflammation effect score. Summing the individual E-DII scores for the 27 food parameters yielded each participant’s overall E-DII score.

# S5. Supplementary method 2: physical activity assessment methods.

In this study, we quantified Total Physical Activity (TPA) utilizing a modified iteration of the International Physical Activity Questionnaire (IPAQ).[2] This assessment entailed a comprehensive record of the frequency and duration of various physical activities (PA), specifically walking, moderate, and vigorous activities, executed over the preceding week. The ensuing data underwent analysis adhering to the established IPAQ scoring protocol. Metabolic Equivalent of Task (MET) values were ascribed to each activity category: walking (3.3 METs), moderate-intensity activities (4.0 METs), and vigorous-intensity activities (8.0 METs).[3] To compute the aggregate weekly PA in MET-minutes per week, we multiplied the documented frequency and duration of the activities by their corresponding MET values. Subsequently, the cohort was stratified into three distinct PA categories based on conventional scoring metrics: low PA (less than 600 MET-minutes/week), moderate PA (600 to less than 3000 MET-minutes/week), and high PA (3000 MET-minutes/week or more). Notably, the threshold of 600 MET-minutes/week aligns with the recommended guidelines advocating for a minimum of 150 minutes of moderate-intensity PA per week.[2]

Objective Physical Activity was meticulously evaluated through the deployment of the Axivity AX3 wrist-worn triaxial accelerometer (Axivity Ltd, Newcastle, UK). Participants were instructed to don the device uninterruptedly over a span of seven days.[4] Those who had furnished a valid email address during the initial assessment were subsequently invited to participate in the accelerometer study, with devices dispatched to their residences post-consent.[5]

In our analysis, we adhered to stringent inclusion criteria, omitting data from participants with insufficient wear time (less than three days),[5] incomplete 24-hour cycle data (n=6,943), failed device calibration (n=162), or PA measurements procured post-mortality registry update (n=121). The mean acceleration value served as a proxy for the cumulative duration engaged in varying PA intensities, a metric previously validated for its strong correlation with total energy expenditure.[6]

The study population was stratified into tertiles based on PA levels: the lowest tertile (<24.0 milli-gravitational units [mg]), the middle tertile (24.0-30.4 mg), and the upper tertile (>30.4 mg). These tertiles were further contextualized by equating them to the duration of activity at or above the intensity of brisk walking (4.3 METs),[3] as per standard PA guidelines. This equivalence was derived from a laboratory calibration study, which established a threshold of 250 mg to correspond with an activity intensity of 4.3 METs.[7] Utilizing Data-Field 90133, we calculated the median (interquartile range) duration of brisk walking for each tertile: the lowest tertile at 4.3 (2.9-7.2) minutes/day, the middle tertile at 10.1 (7.2-14.4) minutes/day, and the upper tertile at 21.6 (15.8-30.2) minutes/day.

For interpretative clarity, objective PA was categorized as low (equivalent to 4 minutes/day of brisk walking), moderate (10 minutes/day), and high (22 minutes/day). Validity of the results was further corroborated through sensitivity analyses confined to the subset of the population exceeding the 250 mg threshold, yielding median values for the lowest, middle, and upper tertiles at 4.3 (2.9-5.8), 11.5 (10.1-14.4), and 24.5 (20.2-31.7) minutes/day of brisk walking, respectively.

# S6. Supplementary method 3: biological age calculation methods.

I. Model Training Using NHANES III Dataset: Initially, we identified 33 potential biomarkers and demographic information available in both NHANES III and the UK Biobank (Supplementary Material: Table S1.5). Only those with data missing ≤20% and correlated with chronological age (|r| > 0.1, based on prior work in NHANES III) were considered. Due to the high correlation between pulse and systolic blood pressure (r = 0.84), pulse was excluded. Consequently, our KDM, PhenoAge, and HD algorithms included 18 biomarkers: waist circumference, FEV1, systolic blood pressure, diastolic blood pressure, blood urea nitrogen, glycated hemoglobin (HbA1c), total cholesterol, creatinine, serum glucose, red cell distribution width, albumin, alkaline phosphatase, triglycerides, mean cell volume, uric acid, lymphocyte percentage, red blood cell count, and C-reactive protein. Following previous research, non-pregnant subjects aged 30-75 years with complete biomarker data were selected as the training population for the KDM computation model (n=9,014). The reference population for PhenoAge included individuals aged 20-84 years with complete biomarker data (n = 12,972). The reference population for HD comprised individuals aged 20-30 years, non-obese, and with biomarkers within the normal range for their age and gender (n = 258).
II. Comparison of New Biological Age Algorithms with Original Algorithms: We assessed the performance of the new biological age algorithms by evaluating their ability to predict mortality and chronological age in an independent cohort of NHANES IV participants recruited during 1999-2014 and followed up until 2015. Similar to the original KDM and PhenoAge algorithms, our new biological age algorithms showed a statistically significant correlation with mortality over a median follow-up time of 7.4 years (interquartile range 4.1-11.5) (Supplementary Material: Table S2.1). Moreover, the new KDM and PhenoAge demonstrated strong correlations with chronological age (r = 0.906, r = 0.957), with all biological age measurements showing moderate correlation in NHANES IV (Supplementary Material: Figures S2.1 and S2.2).
III. Projection of Newly Trained Algorithms onto the UK Biobank: The distribution of biological age measurements in the UK Biobank is depicted in Supplementary Material: Figures S2.4, S2.5, and S2.6.

To calculate the deviation between biological and chronological age, we performed linear regression of KDM and PhenoAge (3 degrees of freedom natural splines) against chronological age, and considered the model residuals as “age residuals” (also termed “age acceleration”). HD did not calculate residuals, as it is not a measure of biological age but a Mahalanobis distance of a set of biomarkers relative to the reference sample (NHANES III). Higher values of KDM residuals, PhenoAge residuals, and HD indicate faster aging. Subsequently, KDM residuals, PhenoAge residuals, and HD were Z-standardized (mean = 0, standard deviation = 1) to facilitate comparison of effect sizes in subsequent analyses.

Reference

1.	Shivappa N, Steck SE, Hurley TG, Hussey JR, Hébert JR: **Designing and developing a literature-derived, population-based dietary inflammatory index**. *Public health nutrition* 2014, **17**(8):1689-1696.

2.	Craig CL, Marshall AL, Sjöström M, Bauman AE, Booth ML, Ainsworth BE, Pratt M, Ekelund U, Yngve A, Sallis JF *et al*: **International physical activity questionnaire: 12-country reliability and validity**. *Medicine and science in sports and exercise* 2003, **35**(8):1381-1395.

3.	Ainsworth BE, Haskell WL, Whitt MC, Irwin ML, Swartz AM, Strath SJ, O'Brien WL, Bassett DR, Jr., Schmitz KH, Emplaincourt PO *et al*: **Compendium of physical activities: an update of activity codes and MET intensities**. *Medicine and science in sports and exercise* 2000, **32**(9 Suppl):S498-504.

4.	**Physical activity monitor (accelerometer)** [<https://biobank.ndph.ox.ac.uk/showcase/ukb/docs/PhysicalActivityMonitor.pdf>]

5.	Doherty A, Jackson D, Hammerla N, Plötz T, Olivier P, Granat MH, White T, van Hees VT, Trenell MI, Owen CG *et al*: **Large Scale Population Assessment of Physical Activity Using Wrist Worn Accelerometers: The UK Biobank Study**. *PloS one* 2017, **12**(2):e0169649.

6.	van Hees VT, Renström F, Wright A, Gradmark A, Catt M, Chen KY, Löf M, Bluck L, Pomeroy J, Wareham NJ *et al*: **Estimation of daily energy expenditure in pregnant and non-pregnant women using a wrist-worn tri-axial accelerometer**. *PloS one* 2011, **6**(7):e22922.

7.	Hildebrand M, VT VANH, Hansen BH, Ekelund U: **Age group comparability of raw accelerometer output from wrist- and hip-worn monitors**. *Medicine and science in sports and exercise* 2014, **46**(9):1816-1824.
